# Two-terminal β-Ga_2_O_3_ photo-synapse for diversified in-sensor computing via self-trapped holes engineering

**DOI:** 10.1038/s41377-026-02298-2

**Published:** 2026-06-29

**Authors:** Yiyin Nie, Shujie Jiao, Xing Yang, Shiyong Gao, Dongbo Wang, Yongfeng Li, Jinzhong Wang, Liancheng Zhao

**Affiliations:** 1 School of Materials Science and Engineering, Harbin Institute of Technology, Harbin, 150001 China; 2 Advanced Laser Technology Laboratory of Anhui Province, Electronic Engineering Institute, National University of Defense Technology, Hefei, 230037 China; 3 Jianghuai Advance Technology Center, Hefei, 230037 China; 4 College of Physics, Jilin University, Changchun, 130012 China

**Keywords:** Optoelectronic devices and components, Photonic devices

## Abstract

The rapid advancement of artificial intelligence has propelled the development of β-Ga_2_O_3_ photo-synapses for solar-blind ultraviolet neuromorphic machine vision systems. However, existing β-Ga_2_O_3_ photo-synapses not only exhibit reduced stability but also display high weight update nonlinearity. Herein, we propose a novel strategy to construct β-Ga_2_O_3_ photo-synapses with low weight update nonlinearity based on self-trapped holes, aiming to achieve multi-level in-sensor computing tasks. Theoretical and experimental investigations revealed that the interaction between the larger effective mass of holes and local lattice distortions in β-Ga_2_O_3_ promoted the formation of self-trapped holes, which significantly reduced hole mobility and enhanced the persistent photocurrent effect. The fabricated β-Ga_2_O_3_ photo-synapses exhibited excellent short-term plasticity, which could be transited to long-term plasticity by adjusting the characteristics of 252 nm ultraviolet light. Moreover, the devices achieved a low weight update nonlinearity of 0.42, outperforming most previously reported photo-synapses. Finally, β-Ga_2_O_3_ photo-synapses were integrated into neuromorphic machine vision systems, enabling tasks ranging from low-level image classification to high-level motion recognition, achieving recognition accuracies of 99.48% and 92.70% on the MNIST and Fashion-MNIST datasets. It also maintained 100% target tracking accuracy under 60% Gaussian noise interference and reached a recognition accuracy of 94.94% for 10 motions in UTD-MHAD dataset. These results highlight great potential of β-Ga_2_O_3_ photo-synapses based on self-trapped holes engineering in the era of artificial intelligence.

## Abstract (web summary)

β-Ga_2_O_3_ photo-synapses based on self-trapped holes engineering exhibited a low weight update nonlinearity of 0.42, thereby enabling multiple machine vision tasks from low-level image classification to high-level motion recognition.

## Graphical abstract

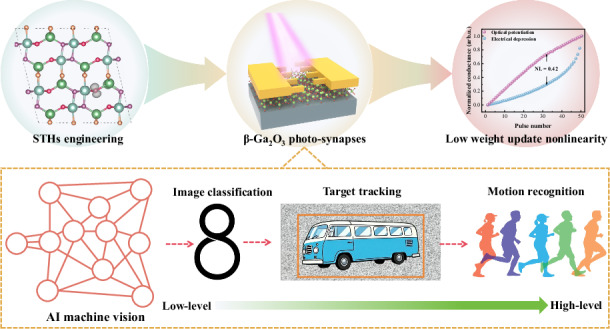

## Introduction

The rapid advancement of artificial intelligence (AI) has significantly accelerated the development of machine vision, particularly in real-time applications such as autonomous driving, where target recognition and classification are essential^1–4^. Neuromorphic machine vision systems based on AI have demonstrated enhanced recognition and processing capabilities. However, these systems still rely on traditional von Neumann architecture, in which sensing, computing, storage, and GPU modules are physically separated^5^. This separation results in high energy consumption, limited signal transmission efficiency, and suboptimal computational performance^6,7^. In contrast, human vision systems achieve remarkable efficiency through pJ-level power consumption and highly parallel processing capabilities^8,9^. Therefore, emulating human vision systems and re-engineering the architecture and hardware components of neuromorphic machine vision systems have emerged as the critical research in this field.

The human visual system can be regarded as a hierarchical information processor composed of retina, optic nerves, and brain, capable of complex visual functions ranging from low-level image classification to high-level motion recognition^10,11^. The retina is responsible for capturing external light signals and performing pre-processing such as filtering, noise reduction, and feature enhancement. Subsequently, optic nerves transmit information to the brain for advanced cognitive functions, including information storage, object recognition, and concept extraction. Neuromorphic machine vision inspired by the human visual system should consist of two components: photo-synapses that integrate sensing and pre-processing, and non-volatile memristors are equipped with storage and computing functions^12,13^. Unlike non-volatile memristors designed for long-term data storage, photo-synapses emphasize real-time sensing and immediate signal pre-processing^14,15^. As ideal in-sensor computing devices, photo-synapses should possess following characteristics: (i) high photoelectric conversion efficiency and rapid detective speed during detection; (ii) after detection, the residual current should decay slowly over time, acting as a temporary data buffer to support in-sensor processing and information transmission; (iii) in certain applications, photo-synapses should also demonstrate a degree of resistance to optical interference^16^. Development of photo-synapses fulfilling these criteria constitutes a fundamental basis for realizing neuromorphic machine vision.

As an ultra-wide bandgap semiconductor, β-Ga_2_O_3_ exhibits excellent electrical properties with a bandgap aligned to the solar-blind ultraviolet region^17–19^. The unique property of β-Ga_2_O_3_ could extend neuromorphic machine vision into solar-blind UV spectrum, offering broad applications such as space exploration and ozone layer imaging^20,21^. The persistent photocurrent (PPC) effect serves as the foundation for β-Ga_2_O_3_ photo-synapses. It is defined by a gradual decay of photogenerated current over time rather than a rapid recombination following the termination of light illumination. Up to date, the majority of reported β-Ga_2_O_3_ photo-synapses have been developed through oxygen vacancy (*V*_O_) engineering to enhance PPC effect^22^. For instance, Long et al. demonstrated two-terminal GaO_x_ photo-synapses through a high density of *V*_O_, which were successfully applied in fingerprint recognition^23^. Recently, Chang et al. exploited *V*_O_ to fabricate Ag/Ga_2_O_3_/Pt resistive random-access memories, which not only exhibited non-volatile characteristics for in-memory computing but also integrated in-sensor computing capability^24^. Nevertheless, PPC effect induced by *V*_O_ involves ionization and migration of *V*_O_ during light exposure. Specifically, *V*_O_ ionize and release electrons under illumination, with *V*_O_ migrating toward the negative electrode and electrons moving toward the positive electrode, leading to the formation of conductive filaments. Upon termination of light exposure, *V*_O_ re-capture electrons. This dynamic process results in a relatively slow detective speed in photo-synapses (Fig. S1,Supplementary Information). Moreover, maintaining a pronounced PPC effect typically requires a number of *V*_O_, which can cause Ga_2_O_3_ to deviate from the ideal stoichiometry, thereby reducing the stability and long-term reliability^25^ (Sentence S1, Supplementary Information). Therefore, developing novel mechanisms for PPC effect represents a critical challenge. In our previous studies, the large effective mass of holes in β-Ga_2_O_3_ was shown to play a crucial role in formation of small polarons, particularly self-trapped holes (STHs), which significantly reduce hole mobility and induce PPC effect (Sentence S2, Supplementary Information)^26–28^. By modulating the concentration of STHs, it is feasible to develop β-Ga_2_O_3_ photo-synapses without *V*_O_ engineering^29,30^.

**Fig. 1:**
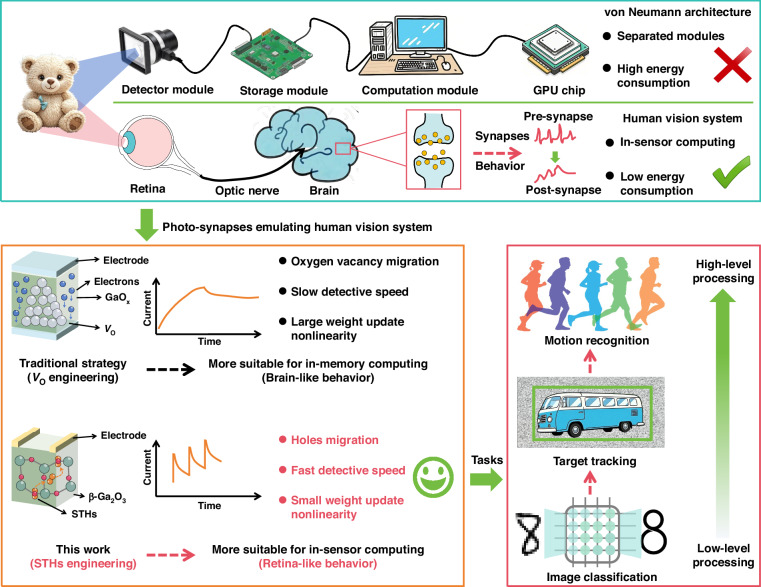
Schematic graph of β-Ga_2_O_3_ photo-synapses for in-sensor computing

In this work, β-Ga_2_O_3_ photo-synapses with anti-interference characteristics and low weight update nonlinearity were successfully fabricated by modulating concentration of STHs. The properties of STHs were elucidated through first-principles calculations, demonstrating the incorporation of local lattice distortions can effectively enhance the concentration of STHs. The mobility of holes is significantly reduced by STHs through the trapped-detrapped process and hopping mechanism, thereby inducing PPC effect. Subsequently, local lattice distortions were successfully induced via atomic peening effect during deposition to enhance the concentration of STHs. β-Ga_2_O_3_ with the highest concentration of STHs exhibited an increase of only 35.61% in rising time and a 4604.92% increase in decay time when responding to 252 nm illumination, which demonstrates that STHs can significantly enhance PPC effect without significantly affecting detective speed, in contrast to *V*_O_ engineering. Based on this, two-terminal β-Ga_2_O_3_ photo-synapses were fabricated with excellent short-term plasticity (STP) and long-term plasticity (LTP). After 50 consecutive optical potentiation and electrical depression stimuli, β-Ga_2_O_3_ photo-synapses had also exhibited a low weight update nonlinearity of 0.42, which is superior to most reported photo-synapses^22,24,31–36^. Furthermore, β-Ga_2_O_3_ photo-synapses were integrated with non-volatile memristors to construct solar-blind ultraviolet neural machine vision system. By leveraging convolutional neural networks (CNN) and reservoir computing (RC) algorithms, β-Ga_2_O_3_ neural machine vision system has demonstrated effective performance in both low-level image classification and high-level motion recognition tasks. It achieved image classification accuracies of 99.48% on the MNIST dataset and 92.70% on the Fashion-MNIST dataset. In the anti-noise target tracking task, the system maintained 100% tracking accuracy even under 60% Gaussian noise interference. Furthermore, it attained a recognition accuracy of 94.94% for 10 types of motions in the UTD-MHAD dataset. This work demonstrated the feasibility of developing high-performance β-Ga_2_O_3_ photo-synapses through STHs engineering, thereby overcoming critical limitations in stability, weight update nonlinearity and response dynamics of β-Ga_2_O_3_ photo-synapses resulting from *V*_O_ strategies. The results highlight the significant potential of β-Ga_2_O_3_ photo-synapses via STHs engineering for advancing neural machine vision in the era of artificial intelligence, as illustrated in Fig. 1.

## Results

### Mechanism of persistent photocurrent effect induced by self-trapped holes

**Fig. 2:**
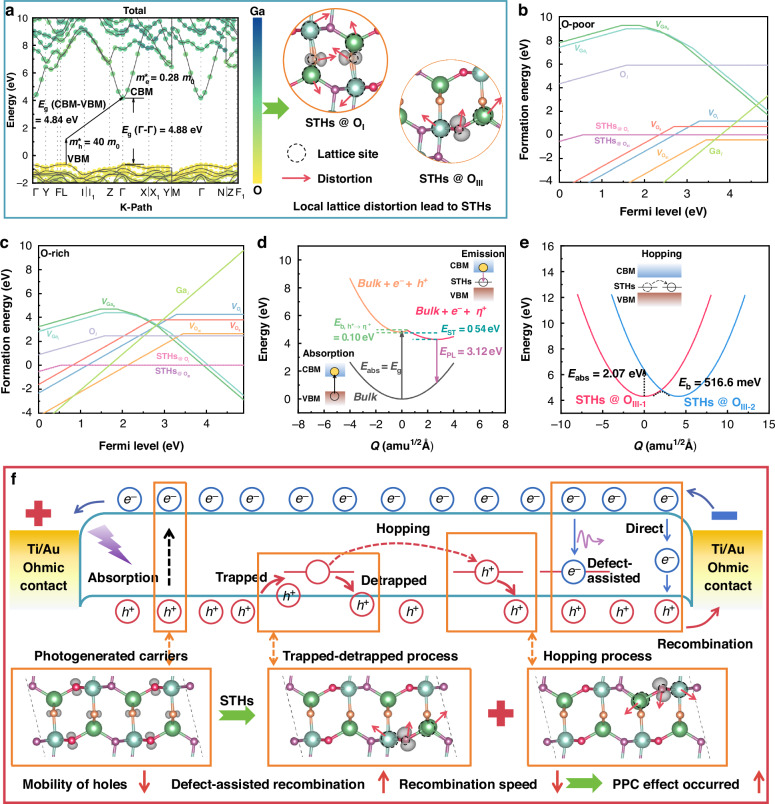
Properties of self-trapped holes in β-Ga_2_O_3_. **a** Formation mechanism of STHs. Formation energy of intrinsic defects of β-Ga_2_O_3_ under **b** O-poor limit and **c** O-rich limit. **d** Trapped-detrapped process of STHs on O_III_ atoms. **e** Configuration coordinate diagrams of hopping process between two neighboring O_III_ atoms. **f** Mechanism of PPC effect induced by STHs

As illustrated in Fig. 2a, conduction-band minimum (CBM) of β-Ga_2_O_3_ is primarily consisted of Ga 4*s* states, resulting in an extremely low effective mass of electrons (${m}_{\mathrm{e}}^{* }$ ≈ 0.28 *m*_0_, as detailed in Sentence S3, Supplementary Information). Meanwhile, valence-band maximum (VBM) along L-I line is predominantly derived from O 2*p* orbitals, characterized by a large effective mass of holes (${m}_{\mathrm{h}}^{* }$ ≈ 40 *m*_0_, Fig. S2, Supplementary Information). Holes with such a large effective mass can be trapped by local lattice distortions, leading to the formation of STHs in β-Ga_2_O_3_ (Fig. S3, Supplementary Information)^37–39^. In defect-free β-Ga_2_O_3_, STHs are distributed between two distorted O_I_ atoms by perturbing four adjacent Ga atoms. Furthermore, STHs can also form at a single O_III_ site by inducing local lattice distortions in two neighboring Ga atoms (Figs. S4 and S5, Supplementary Information). The formation energies of intrinsic defects under oxygen-poor limit (O-poor) and oxygen-rich limit (O-rich) in β-Ga_2_O_3_ were shown in Fig. 2b, 2c, respectively. Compared with other intrinsic defects, the formation energies of STHs remain stable, indicating STHs are primarily influenced by local lattice distortions rather than growth environment. The thermodynamic transition levels of ${{{\rm{STHs}}}_{{\rm{@O_I}}}}$ and ${{{\rm{STHs}}}_{{\rm{@O_{III}}}}}$ were 0.56 eV and 0.54 eV, respectively (Table S1, Supplementary Information), confirming that the STHs were widely existed in defect-free β-Ga_2_O_3_.

STHs in β-Ga_2_O_3_ influence the mobility of holes through both trapped-detrapped process and hopping mechanism. To simplify the discussion, this work focuses solely on trapped-detrapped process and hopping mechanism of ${\mathrm{STHs}}_{\mathrm{@}{\mathrm{O}}_{{\rm{III}}}}$, with similar conclusions are expected for ${\mathrm{STHs}}_{\mathrm{@}{\mathrm{O}}_{{\rm{I}}}}$. After absorption of ultraviolet light, photogenerated electrons and holes are created in β-Ga_2_O_3_ (Figs. 2d and S6 in Supplementary Information). Then, the photogenerated holes can be trapped into the ${\mathrm{STHs}}_{\mathrm{@}{\mathrm{O}}_{{\rm{III}}}}$ by overcoming a small barrier, ${{E}_{{\rm{b}},{\rm{h}}^+\to{\upeta}^+}}=0.10$ eV, known as trapped process. Conversely, the trapped hole can be delocalized by overcoming a large barrier, ${{E}_{{\rm{b}},{\upeta}^+\to{\rm{h}}^+}}={{E}_{{\rm{b}},{\rm{h}}^+\to{\upeta}^+}}+{{E}}_{\rm{ST}}=0.64$ eV, referred to detrapped process. The significant disparity in energy barriers between trapping and detrapping results in a stronger tendency for photogenerated holes to remain localized around oxygen atoms, thereby substantially reducing their overall mobility^40^.

Additionally, the trapped holes can also migrate via hopping mechanism (Figs. 2e and S7 in Supplementary Information). According to Marcus’ theory, the trapped holes in ${\mathrm{STHs}}_{\mathrm{@}{\mathrm{O}}_{{\rm{III}}}}$ are excited out of their self-trapping potential well and subsequently hop to another neighboring ${\mathrm{STHs}}_{\mathrm{@}{\mathrm{O}}_{{\rm{III}}}}$ with the assistance of photoexcitation^41^. The migration barrier (${E}_{\mathrm{b}}$) can be calculated using coordinates of intermediate configurations, defined as $R=x{R}_{\mathrm{i}}+(1-x){R}_{\mathrm{f}}$, where $R$, ${R}_{\mathrm{i}}$ and ${R}_{\mathrm{f}}$ represent coordinates of all atoms in β-Ga_2_O_3_ associated with intermediate, initial and final configurations, $x$ is interpolation parameter for intermediate configuration (Fig. S8, Supplementary Information). For ${\mathrm{STHs}}_{\mathrm{@}{\mathrm{O}}_{{\rm{III}}}}$, ${E}_{\mathrm{b}}$ was found to be 516.6 meV and the related absorption energy can be estimated as ${E}_{\mathrm{abs}}=4{E}_{\mathrm{b}}=2.07{\rm{eV}}$. The hopping mobility (${\mu }_{\mathrm{hopping}}$) of ${\mathrm{STHs}}_{\mathrm{@}{\mathrm{O}}_{{\rm{III}}}}$ can be approximated as ${\mu }_{\mathrm{hopping}}=[e{a}^{2}{\omega }_{0}/{k}_{B}T][\exp (-{E}_{\mathrm{b}}/{k}_{B}T)]$, where $a=3.80\,{\mathrm{\AA}}$ is hopping distance and ${\omega }_{0}$ is longitudinal phonon frequency^41^. Such a large ${E}_{\mathrm{b}}$ results in a lower ${\mu}_{\rm{hopping}}$ at room temperature, implying that the photogenerated holes captured by STHs can still migrate through hopping mechanisms. However, the overall mobility will be significantly reduced.

When ultraviolet light irradiates β-Ga_2_O_3_ in Fig. 2f, photogenerated electrons and holes are generated and migrate toward the electrodes under bias. After illumination, the photogenerated carriers must migrate to specific regions to recombine. However, the mobility of holes is significantly reduced due to STHs, resulting in the prolonged migration time for holes. In contrast, the mobility of electrons is almost unaffected. Consequently, a large number of photogenerated electrons and holes fail to recombine and remain in the circuit, thereby giving rise to PPC effect. Moreover, electrons can also recombine with holes via STHs-assisted mechanisms, emitting 3.12 eV ultraviolet light as they return to the ground state. However, this defect-assisted recombination is less efficient compared to direct recombination, further decreasing the carrier recombination rate and amplifying PPC effect. Therefore, increasing the concentration of STHs can effectively enhance PPC effect in β-Ga_2_O_3_ providing a foundation of photo-synaptic applications.

### Modulation of self-trapped holes in β-Ga_2_O_3_ films

**Fig. 3:**
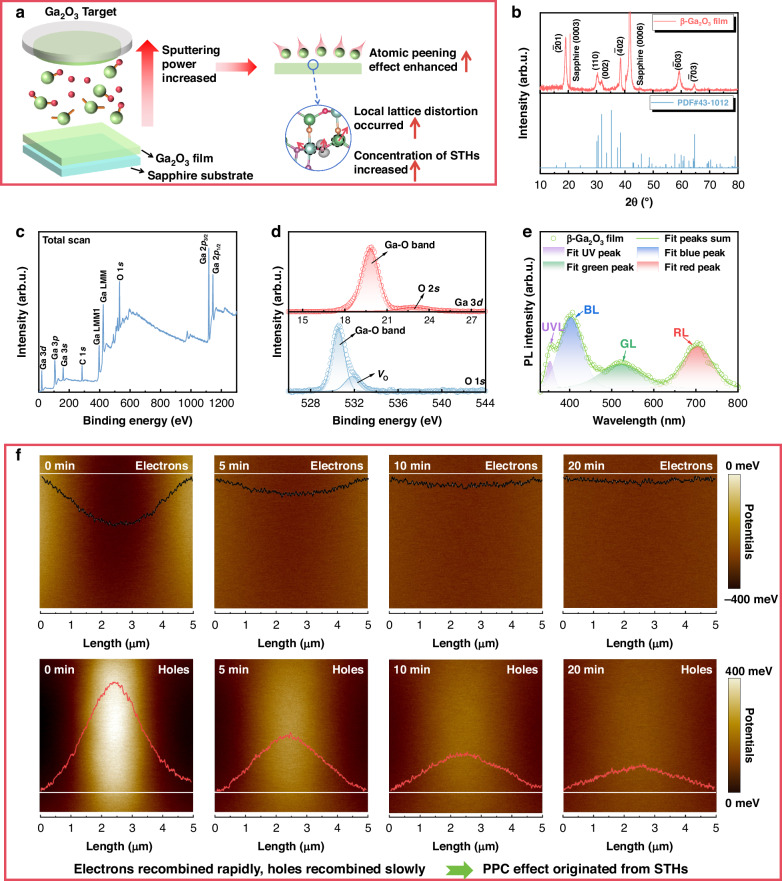
Preparation and film-analysis of β-Ga_2_O_3_. **a** Increasing STHs by atomic peening effect. **b** XRD, **c** XPS total scan, **d** Ga 3*d* and O 1*s*, **e** PL spectrum, **f** KPFM of β-Ga_2_O_3_ surface after injecting electrons and holes

As shown in Fig. 3a, β-Ga_2_O_3_ films were deposited by increasing sputtering power to enhance atomic peening effect, thereby inducing local lattice distortion during deposition (Table S2, Supplementary Information). Optical bandgap and thickness of β-Ga_2_O_3_ films are provided in Figs. S9 and S10 (Supplementary Information). XRD pattern are shown in Fig. 3b and Fig. S11 (Supplementary Information). Three distinct diffraction peaks appeared at approximately 19.02°, 38.42° and 59.43 ° are identified as $(\bar{2}01)$, $(\bar{4}02)$ and $(\bar{6}03)$ peaks of $(\bar{2}01)$ plane family in β-Ga_2_O_3_^42^. Three weaker diffraction peaks observed at approximately 30.12°, 31.83° and 64.60° corresponding to $(110)$, $(002)$ and $(\bar{7}03)$ planes (JCPDS CARD No. 76-0573), emerged when sputtering power was greater than 100 W (Fig. S11, Supplementary Information). XPS (Figs. 3c, d, S12 and S13 in Supplementary Information) and EDS (Fig. S14, Supplementary Information) were employed to analyze elemental composition of β-Ga_2_O_3_ films. No additional elements beyond Ga and O were detected, indicating β-Ga_2_O_3_ films possess high purity. The ratio of Ga and O atoms in Table S3 (Supplementary Information) are close to ideal stoichiometric ratio and remain relatively consistent. The percentage of *V*_O_ in Figs. 3d and S13 (Supplementary Information) also remained relatively consistent (Table S4, Supplementary Information), indicating that increased sputtering power did not significantly modify growth environment of β-Ga_2_O_3_ films and has minimal impact on the concentration of defects associated with Ga and O atoms (*V*_Ga_, *V*_O_, Ga_*i*_ and O_*i*_), which is in good agreement with DFT calculations.

The concentration of STHs can be qualitatively characterized by relative intensity of their PL spectrum (Fig. 3e, Sentence S4, Figs. S6 and S15 of Supplementary Information). PL spectrum exhibits four distinct peaks, which can be categorized as ultraviolet luminescence (UVL), blue luminescence (BL), green luminescence (GL) and red luminescence (RL). BL is attributed to ${{(V}_{\mathrm{O}}+{V}_{\mathrm{Ga}})}^{-}$ and GL originates from isolate ${V}_{\mathrm{Ga}}^{2-}$, representing the concentration of intrinsic Ga-related/O-related defects^26^. RL is associated with unintentional doping by nitrogen atoms^26,43^. The UVL, in contrast, is generated from STHs. The relative intensities of these four PL peaks were listed in Table S5 (Supplementary Information). As sputtering power increased from 60 W to 120 W, the relative intensity of UVL enhanced accordingly, indicating the concentration of STHs reached its maximum when sputtering power was 120 W (Sentence S5, Supplementary Information). To verify the impact of STHs on hole mobility, Kelvin probe force microscopy (KPFM) was employed to investigate the surface potential distribution of β-Ga_2_O_3_ films, as shown in Fig. 3f and Fig. S16 (Supplementary Information). In the target regions (5 × 5 μm²), extrinsic electrons or holes were injected exclusively into the central areas (1 × 5 μm²). Following carrier injection, the dynamic evolution of surface potential distributions and height within target regions was monitored at 5 min intervals.

As illustrated in Fig. 3f, the decay rate of electron potential on the surface of β-Ga_2_O_3_ film grown at 120 W was significantly higher than that of hole potential. The electron potential decayed to baseline within 10 min, whereas the hole potential retained a residual value of 88.15 meV even after 20 min. These results suggest that the β-Ga_2_O_3_ film fabricated at 120 W exhibits a high concentration of STHs, which effectively suppress hole mobility and result in a longer migration time to recombination sites. The same KPFM measurement was performed on the β-Ga_2_O_3_ film grown at 60 W in Fig. S17 (Supplementary Information). Although the difference in ${m}_{\mathrm{e}}^{* }$ and ${m}_{\mathrm{h}}^{* }$ of β-Ga_2_O_3_ may influence their mobility, both the electron potential and hole potential in Fig. S17 (Supplementary Information) decayed to baseline within 10 minutes. Thus, the results in Fig. 3f can be attributed to the inhibitory effect of STHs on hole mobility, rather than the difference in ${m}_{\mathrm{e}}^{* }$ and ${m}_{\mathrm{h}}^{* }$ of β-Ga_2_O_3_. β-Ga_2_O_3_ films fabricated at 120 W sputtering power exhibited highest concentration of STHs and lowest hole mobility, thereby inducing the PPC effect and enabling the realization of photo-synapses.

### Performance of two-terminal β-Ga_2_O_3_ photo-synapses

**Fig. 4:**
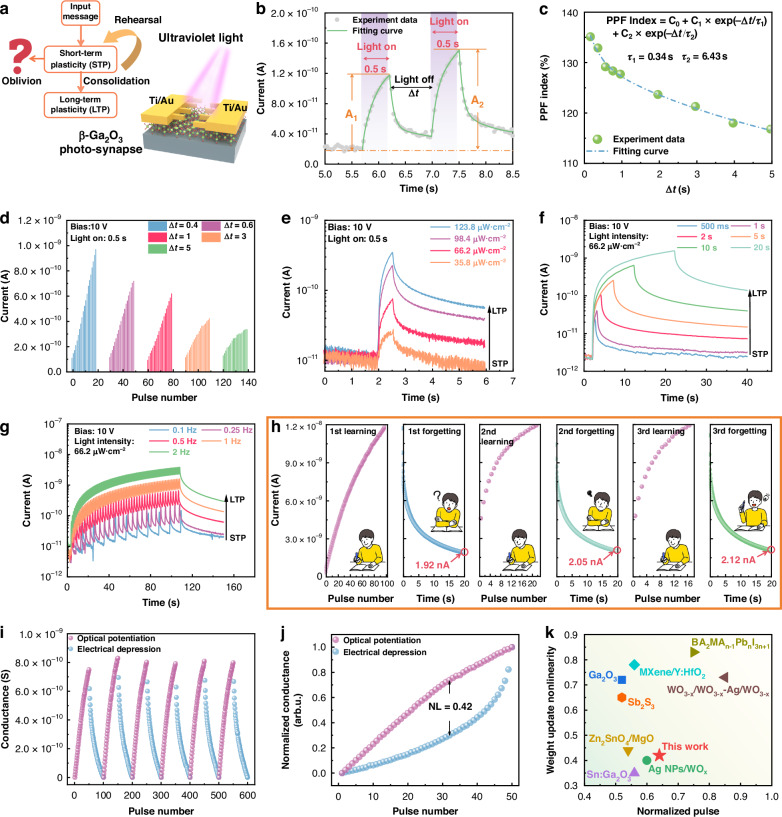
Performance of two-terminal β-Ga_2_O_3_ photo-synapses. **a** Schematic graph of two-terminal β-Ga_2_O_3_ photo-synapses. **b** PPF behavior of β-Ga_2_O_3_ photo-synapses triggered by a pair of successive optical pulses (∆*t* = 800 ms). **c** PPF index as a function of ∆*t*. **d** Spike rate dependent plasticity of β-Ga_2_O_3_ photo-synapses. STP-to-LTP transition induced by **e** light intensity, **f** duration time, **g** frequencies. **h** Learning-experience behavior of β-Ga_2_O_3_ photo-synapse. **i** Long-term conductance under UV optical potentiations and electronic depressions. **j** Weight update nonlinearity under the optical potentiation and electrical depression behaviors. **k** Summary of weight update nonlinearity of various photo-synapses^22,24,31–36^

β-Ga_2_O_3_ film prepared at the sputtering power of 120 W exhibited highest concentration of STHs and demonstrated a pronounced PPC effect, making it suitable for the fabrication of photo-synapses employed in solar-blind UV region. The device structure of β-Ga_2_O_3_ photo-synapses is shown in Figs. 4a and S18 (Supplementary Information). Ti/Au electrodes were optimized in our previous study^27,28^. The electrode parameters are listed in Table S6 (Supplementary Information). Response peak of β-Ga_2_O_3_ photo-synapse appeared at 252 nm (Fig. S19, Supplementary Information). Meanwhile, rising time (*τ*_r_) increased from 2.05 s to 2.78 s, representing the 35.61% increase. The decay time (*τ*_d_) increased from 109.46 ms to 5.15 s, corresponding to the 4604.92% increase (Fig. S19, Supplementary Information). This result not only demonstrated that PPC effect induced by STHs did not degrade the detection speed of β-Ga_2_O_3_ photo-synapses, highlighting a distinct advantage over *V*_O_ strategy, but also provided direct experimental evidence that the synaptic behavior observed in our β-Ga_2_O_3_ photo-synapses primarily originated from STHs rather than from the oxygen vacancies in the film (Fig. S20, Sentence S6, Supplementary Information)^44,45^.

The fundamental synaptic functions of β-Ga_2_O_3_ photo-synapses need to be verified, such as STP, LTP, and learning-experience behavior. Pair-pulse facilitation (PPF) in Fig. 4b, c and Fig. S21 (Supplementary Information) is a well-known phenomenon that reflect STP behavior. This was achieved by changing time interval (∆*t*) between two consecutive 252 nm light pulses from 200 ms to 5 s, while maintaining a light intensity of 66.2 μW cm^−2^ and an exposure time of 500 ms under a 10 V bias. PPF index can be represented by ${\rm{PPF}} \,{\rm{index}}\,=\,{\rm{C}}_{0}+{\rm{C}}_{1}\,{\times}\,{\exp}\,(-\frac{{\triangle}{t}}{{\tau}_{1}})+{\rm{C}}_{2}\,{\times}\,{\exp}\,(-\frac{{\triangle}{t}}{{\tau}_{2}})$, where $\triangle t$ signifies time interval between light pulses, ${\rm{C}}_{0}$, ${\rm{C}}_{1}$ and ${\rm{C}}_{2}$ correspond to initial facilitation magnitudes^46^. PPF index is determined by calculating the ratio of the enhanced excitatory postsynaptic current (EPSC) evoked by the second stimulus (A_2_) to that evoked by the first one (A_1_), ${\tau }_{1}$ and ${\tau }_{2}$ represent characteristic relaxation time for the rapid and slow decay phases. As shown in Fig. 4c, ${\tau }_{2}$ = 6.43 s is one order of magnitude larger than ${\tau }_{1}$ = 0.34 s, which is the characteristic of successive synaptic stimulation^46,47^. Spike trains can extend PPF function into spike-rate-dependent plasticity (SRDP) function as shown in Fig. 4d. Each spike train consists of 20 consecutive ultraviolet spikes (66.2 μW cm^−2^, pulse width of 500 ms). The spike rate was modulated by varying ∆*t* from 0.4 s to 5 s. Similar to the SRDP function observed in biological synapses, an increase in spike rate leads to significantly enhanced EPSC.

By adjusting the intensity, duration time, and frequency of ultraviolet light pulses, the transition from STP to LTP can be effectively induced in β-Ga_2_O_3_ photo-synapses in Fig. 4e–g. As light intensity increased from 35.8 μW cm^−2^ to 123.8 μW cm^−2^ (Fig. 4e), the peak current rose from 28.0 pA to 34.52 pA. After illumination, residual current after 3.5 s decay time increased from 5.75 pA to 56.98 pA, suggesting a transition from STP to LTP in β-Ga_2_O_3_ photo-synapse. Similar phenomenon could be observed by changing duration time and frequency of light stimuli. The peak current rose from 23.54 pA to 1545.48 pA and the residual current increased from 2.48 pA to 138.44 pA in Fig. 4f. As depicted in Fig. 4g, the peak current increased from 0.15 nA to 3.49 nA with elevated light stimulus frequency, while residual current after a 40 s decay period increased from 19.71 pA to 294.32 pA. These results demonstrate that β-Ga_2_O_3_ photo-synapses exhibit the excellent light-dependent multi-storage memory model, where STP state could transition to LTP state, allowing for the long-term storage of information. The function of β-Ga_2_O_3_ photo-synapses in simulating the learning-experience behavior of human brain is presented in Fig. 4h and Fig. S22 (Supplementary Information). In the 1st learning process, the application of 100 light pulses to the device resulted in a current increase to 11.82 nA. Subsequently, during the 20 s decay period, the 1st forgetting process happened, with the current gradually decaying to 1.92 nA, rather than immediately returning to the initial value. In the subsequent second and third cycles, fewer light pulses stimuli (22 pulses and 17 pulses) were applied to β-Ga_2_O_3_ photo-synapse to elicit an identical photocurrent (≥11.82 nA). Additionally, larger decay currents were observed during the forgetting periods of 20 s, leading to 2.05 nA for 2nd forgetting process and 2.12 nA for 3rd forgetting process.

Stability is a critical performance metric that significantly influences the practicality of β-Ga_2_O_3_ photo-synapses. By utilizing 252 nm ultraviolet light pulses for optical potentiation and −10 V bias pulses for electrical depression, synaptic current can be modulated as depicted in Figs. 4i and S23 (Supplementary Information), 50 optical potentiation pulses and 50 electrical depression pulses were sequentially applied to β-Ga_2_O_3_ photo-synapses to evaluate their cyclic stability. After repeating the stimulation cycle six times, no significant degradation in performance was observed, demonstrating that β-Ga_2_O_3_ photo-synapses exhibited excellent cyclic stability. The storage stability of β-Ga_2_O_3_ photo-synapses is illustrated in Fig. S24 (Supplementary Information). The results indicated that after 24 months of storage under ambient conditions, the performance of β-Ga_2_O_3_ photo-synapses degraded by only 1.20%. To evaluate the reproducibility of β-Ga_2_O_3_ photo-synapses based on STHs engineering, eight β-Ga_2_O_3_ photo-synapses were fabricated by the same process. The learning-experience behaviors were measured across all devices. The results revealed the high level of consistency in synaptic behavior, demonstrating excellent device-to-device reproducibility (Fig. S25, Supplementary Information). These findings indicated that β-Ga_2_O_3_ photo-synapses based on STHs engineering exhibited not only outstanding storage stability but also robust reproducibility.

Linearity and symmetry of weight update significantly influence the recognition accuracy of photo-synapses in neuromorphic computing systems. The weight update nonlinearity (NL) evolution can be calculated by $\mathrm{NL}=\frac{\max \left|{\mathrm{G}}_{\mathrm{P}}^{\mathrm{i}}-{\mathrm{G}}_{\mathrm{D}}^{\mathrm{i}}\right|}{{\mathrm{G}}_{\max }-{\mathrm{G}}_{\min }}$, where ${\mathrm{G}}_{\mathrm{P}}^{\mathrm{i}}$ and ${\mathrm{G}}_{\mathrm{D}}^{\mathrm{i}}$ represent the conductance of β-Ga_2_O_3_ photo-synapses under the i-th pulse stimulation during the optical potentiation and electrical depression. ${\mathrm{G}}_{\max }$ and ${\mathrm{G}}_{\min }$ denote maximum conductance after 50 pulses and the minimum conductance observed in initial state, respectively (Fig. S26 and Fig. S27 of Supplementary Information)^48^. As shown in Fig. 4j, the NL value of β-Ga_2_O_3_ photo-synapses reached 0.42 at the 32nd pulse. Compared with previously reported photo-synapses in Fig. 4k, the β-Ga_2_O_3_ photo-synapses in this work exhibited a lower NL value and improved symmetry.

As mentioned in Fig. S1 (Supplementary Information), β-Ga_2_O_3_ photo-synapses based on *V*_O_ strategy belong to conductive filamentary photo-synapses, which modulate conductivity via ionized *V*_O_. This implies that the conductivity change arises from migration of ionized *V*_O_ and the transport of photogenerated carriers. Owing to the relatively sluggish migration kinetics of ionized *V*_O_, only a fraction of the defect-induced conductivity increment is collected by electrodes under illumination. Consequently, only a portion of the total induced conductivity change is effectively extracted under linear optical potentiation. As a result, the β-Ga_2_O_3_ photo-synapses based on *V*_O_ strategy exhibit the large weight update nonlinearity. In contrast, β-Ga_2_O_3_ photo-synapses in this work belong to charge trapping photo-synapses, whose synaptic behavior results exclusively from carrier trapping during transport, instead of defects migration. Consequently, generation and recombination of photocurrent occurred more rapidly, leading to the more linear conductance response and the lower weight update nonlinearity than that of the conventional *V*_O_-based β-Ga_2_O_3_ photo-synapses. Furthermore, β-Ga_2_O_3_ photo-synapses exhibited outstanding low-power performance, as shown in Table S7 (Supplementary Information). These results demonstrate that β-Ga_2_O_3_ photo-synapses optimized via STHs engineering exhibited enhanced synaptic performance, offering robust support for a wide range of neuromorphic in-sensor computing applications (Tables S8 and S9, Supplementary Information).

### Image classification by β-Ga_2_O_3_ photo-synapses based in-sensor computing system

**Fig. 5:**
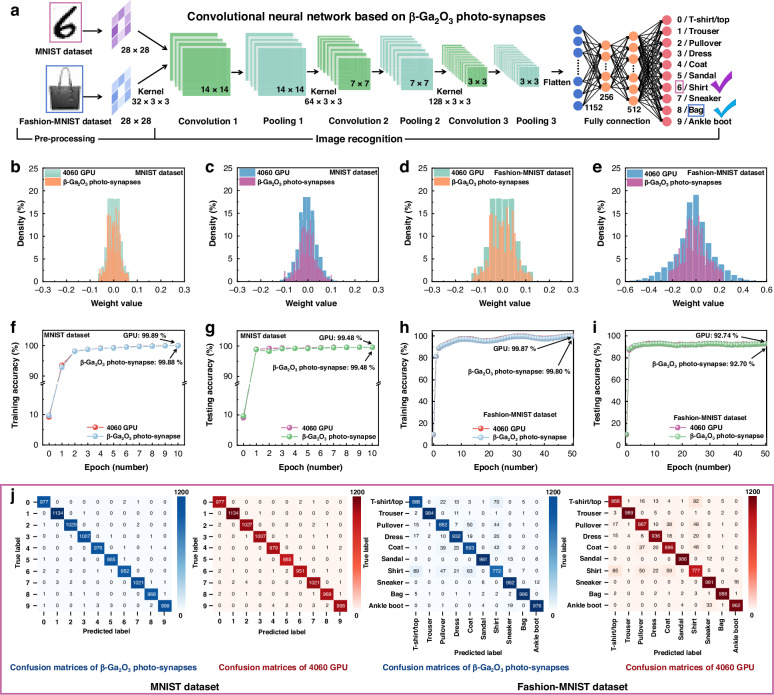
Image classification based on β-Ga_2_O_3_ photo-synapses. **a** Schematic graph of convolutional neural network based on β-Ga_2_O_3_ photo-synapses. **b** Initial weight values and **c** final weight values of 4060 GPU and β-Ga_2_O_3_ photo-synapses in MNIST dataset training. **d** Initial weight values and **e** final weight values of 4060 GPU and β-Ga_2_O_3_ photo-synapses in Fashion-MNIST dataset training. **f** Training accuracy and **g** testing accuracy of 4060 GPU and β-Ga_2_O_3_ photo-synapses in MNIST dataset. **h** Training accuracy and **i** testing accuracy of 4060 GPU and β-Ga_2_O_3_ photo-synapses in Fashion-MNIST dataset. **j** Confusion matrices of 4060 GPU and β-Ga_2_O_3_ photo-synapses in MNIST dataset and Fashion-MNIST dataset

Classification of images is a critical function in low-level visual processing within human visual system. In this work, leveraging β-Ga_2_O_3_ photo-synapses with Convolutional Neural Network (CNN) algorithm enabled the successful recognition and classification of both MNIST dataset and Fashion-MNIST dataset in Fig. 5a. Both datasets consist of 28 × 28-pixel grayscale images of 10 categories with each dataset containing 60,000 training images and 10,000 testing images^48^. The 28 × 28 pixel information of input images were progressively extracted and transformed into 1152 feature values through three stages of convolution and pooling. These features were sequentially processed by two hidden layers comprising 256 and 512 neurons, respectively. The 10 neurons in the output layer corresponded to the 10 distinct categories in the MNIST and Fashion-MNIST datasets, enabling the classification of images.

During computation, the weight variations of β-Ga_2_O_3_ photo-synapses were determined by conductance changes as depicted in Fig. 4j. In parallel, the same algorithm was evaluated using an NVIDIA RTX 4060 GPU (referred to as the 4060 GPU). The evolution of weight changes in MNIST and Fashion-MNIST datasets is presented in Fig. 5b–e. It is evident that weight distribution transitioned from an initially concentrated state to a symmetric distribution upon completion of model training, regardless of whether CNN algorithm was implemented using β-Ga_2_O_3_ photo-synapses or 4060 GPU.

As shown in Fig. 5f–i, the accuracy of 4060 GPU on MNIST training and test sets reached 99.89% and 99.48% after 10 training epochs. β-Ga_2_O_3_ photo-synapses achieved corresponding accuracy of 99.88% and 99.48%. Given the more complex visual features in the Fashion-MNIST dataset, the training time was increased to 50 epochs. The 4060 GPU attained accuracy of 99.87% on the training set and 92.74% on the test set, whereas β-Ga_2_O_3_ photo-synapses achieved 99.80% and 92.70%, respectively. The classification performance of CNN models trained using 4060 GPU and β-Ga_2_O_3_ photo-synapses across different image categories is presented in Fig. 5j. These results demonstrated that our β-Ga_2_O_3_ photo-synapses exhibited excellent synaptic behavior, enabling CNN-based models to achieve recognition accuracy comparable to that of commercial GPUs (Sentence S7, Supplementary Information).

### Anti-noise interference object tracking based on β-Ga_2_O_3_ photo-synapses

**Fig. 6:**
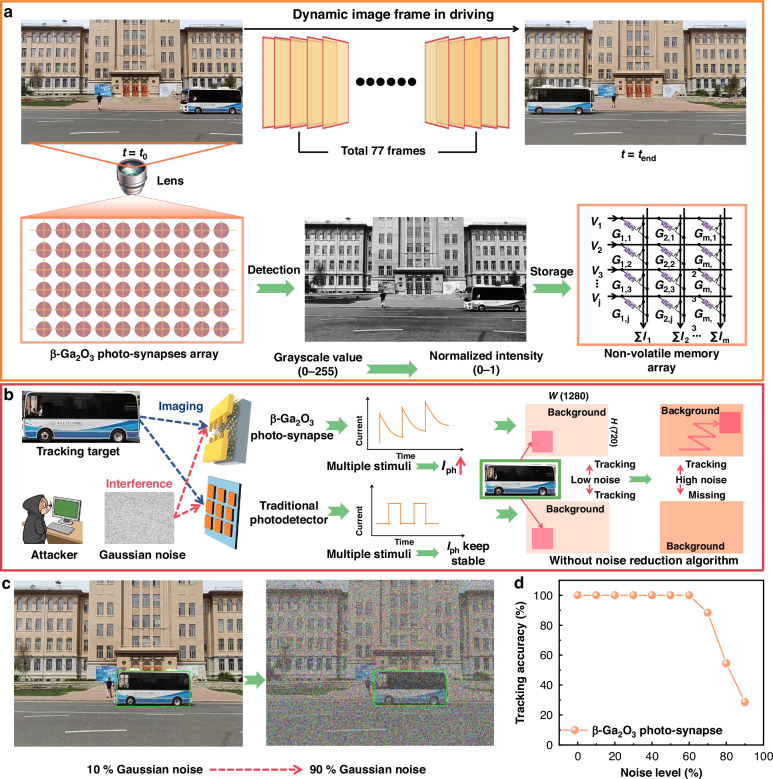
Object tracking based on β-Ga_2_O_3_ photo-synapses. **a** Schematic graph of object tracking based on β-Ga_2_O_3_ photo-synapses. **b** Difference in noise resistance between traditional photodetectors and β-Ga_2_O_3_ photo-synapse based in-sensor computing system in object tracking. **c** Object tracking results of β-Ga_2_O_3_ photo-synapse based in-sensor computing system with various background Gaussian noises. **d** Tracking accuracy of β-Ga_2_O_3_ photo-synapse based in-sensor computing system under various background Gaussian noise level

β-Ga_2_O_3_ photo-synapses exhibit learning-experience behavior analogous to that of human brain. Upon repeated perception of the same target, they produce enhanced output current, thereby demonstrating a certain level of anti-interference capability. This characteristic eliminates the necessity for noise reduction algorithms when employed in target tracking applications. As illustrated in Fig. 6a, a visual perception system was designed using β-Ga_2_O_3_ photo-synapses. A 77-frame video with a resolution of 1280 × 720 dpi was employed, where the target was a moving bus. An array composed of 1280 × 720 virtual photo-synapses was developed by Python code, with each virtual photo-synapses exhibiting learning-experience characteristics consistent with those of β-Ga_2_O_3_ photo-synapses in this work. Since β-Ga_2_O_3_ photo-synapses are insensitive to visible light, the video was pre-processed into grayscale images, where grayscale value corresponded to 252 nm ultraviolet light intensity in a range of 0–1. The output currents of photo-synapses were stored in non-volatile memristors. Given that grayscale value of bus window is 255, corresponding to the maximum normalized light intensity, object tracking was achieved by analyzing distribution of peak output currents.

Throughout tracking process, no noise reduction algorithms were implemented. Instead, Gaussian noise was incrementally introduced to video at 10% intensity intervals. Figure 6b exhibited differences between the imaging systems based on traditional photodetectors and β-Ga_2_O_3_ photo-synapses which subjected to noise interference. The attacker misled the systems by applying a wide range of random interfering light, which can be represented in the video as Gaussian noise and increase background light intensity. Traditional photodetectors clear photocurrent after each perception, ensuring photocurrent obtained in next perception is the same as previous one. Since light intensities of background and the perceived target remained unchanged, traditional photodetectors rely on the photocurrent contrast generated by the difference in light intensity between background and the target to achieve target tracking. However, when Gaussian noise caused light intensity of background to gradually approach that of target, traditional photodetectors without the noise reduction algorithm would lose photocurrent contrast, resulting in the loss of target.

In contrast, the photocurrent of β-Ga_2_O_3_ photo-synapses did not immediately clear after each perception but decayed slowly. In the next perception, the photocurrent was higher than previous one, gradually enhancing the signal of target. Although background light intensity also increased, the photocurrent difference between background and target always existed, due to the time accumulation required for the influence of Gaussian noise on β-Ga_2_O_3_ photo-synapses. This allows the system to maintain continuous contrast for target tracking in Fig. 6b–d. The noise-resistant performance of β-Ga_2_O_3_ photo-synapses system was illustrated in Fig. 6d. When the intensity of Gaussian noise was below 60%, β-Ga_2_O_3_ photo-synapses achieved 100% tracking accuracy. As the intensity of Gaussian noise was further increased, the tracking accuracy gradually declined (Table S10 and Movie S1, Supplementary Information). Remarkably, even under 90% Gaussian noise, β-Ga_2_O_3_ photo-synapses still maintained 28.6% tracking accuracy, demonstrating its strong inherent capability to resist interference (Sentences S8 and S9, Supplementary Information).

### Motion recognition through reservoir computing by β-Ga_2_O_3_ photo-synapses

**Fig. 7:**
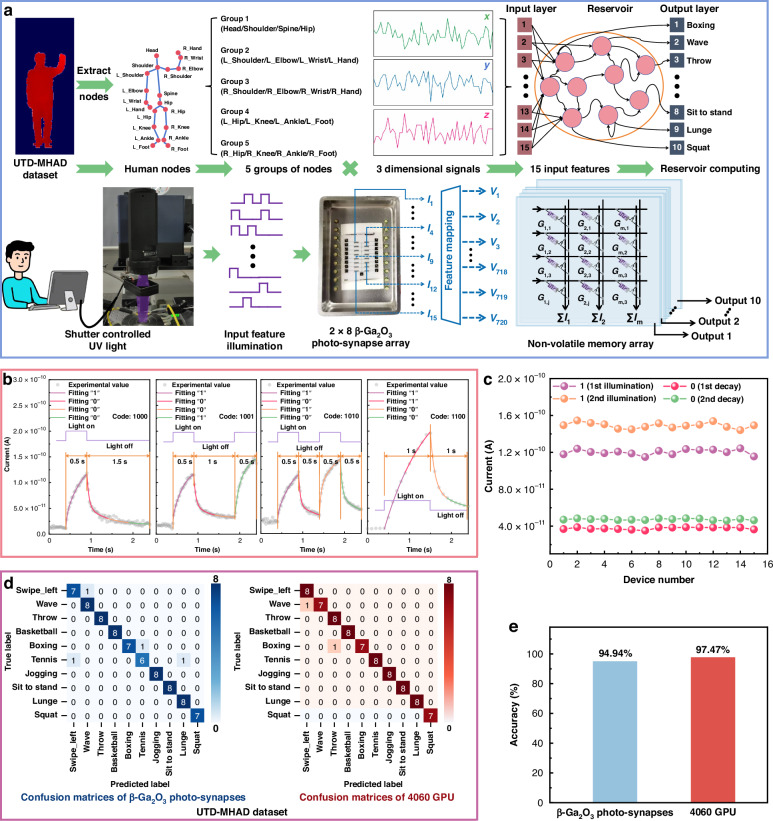
Motion recognition based on β-Ga_2_O_3_ photo-synapses. **a** Schematic graph of motion recognition based on β-Ga_2_O_3_ photo-synapses. **b** “1000”, “1001”, “1010”, and “1100” states of β-Ga_2_O_3_ photo-synapses. **c** Current values of β-Ga_2_O_3_ photo-synapse array in “1010” state. **d** Confusion matrices of 4060 GPU and β-Ga_2_O_3_ photo-synapse array. **e** Motion recognition accuracy of 4060 GPU and β-Ga_2_O_3_ photo-synapse array in UTD-MHAD dataset

Reservoir computing (RC) demonstrates superior capability in handling time series tasks that traditional neural networks often struggle to model effectively. Furthermore, RC requires training only the connection weights of output layer, thereby significantly reducing computational complexity^49^. ESPC of β-Ga_2_O_3_ photo-synapses presents a high degree of compatibility and promising application prospects for the physical implementation of RC. As demonstrated in Fig. 7a, a physical RC system was implemented by 2 × 8 β-Ga_2_O_3_ photo-synapses array, in which β-Ga_2_O_3_ photo-synapses functioned as virtual dynamic sensor reservoir nodes, while non-volatile memristors served as read-out layer. This architecture enabled accurate classification of 10 actions from UTD-MHAD dataset (Sentence S10, Supplementary Information)^50^. To process complex human motion signals, skeleton nodes are aggregated into 5 body groups, and 3D coordinates are extracted to generate 15 parallel feature streams, which are used to stimulate 15 synapses. The device’s non-linear photocurrent decay is leveraged to implement a virtual node strategy through time-multiplexing: the transient response of each device is sampled at 48 discrete time steps. This physically expands the 15 low-dimensional inputs into 720 high-dimensional spatiotemporal features. These feature currents are subsequently converted to voltage signals and applied to the non-volatile memristor array for physical weighted summation, enabling efficient classification of the 10 human actions. The features were input in groups of 4 bits, forming 16 distinct encoding patterns ranging from “0000” to “1111”. The corresponding *I*-*t* curves were presented in Fig. 7b (Sentence S11, Sentence S12, Fig. S28, Supplementary Information).

To evaluate uniformity of 15 β-Ga_2_O_3_ photo-synapses, *I*-*t* curves of “1010” encoded light stimulus was applied in Fig. 7c. The results exhibited strong consistency among the photo-synapses. For comparison, the same algorithm was executed on 4060 GPU. Confusion matrices and accuracy were generated as shown in Fig. 7d, e. For UTD-MHAD dataset, RC algorithm on 4060 GPU achieved an accuracy of 97.47%, whereas β-Ga_2_O_3_ photo-synapses based RC system attained an accuracy of 94.94%. These results demonstrate that β-Ga_2_O_3_ photo-synapses exhibit robust performance and hold significant potential for application in RC systems.

## Discussion

In this work, β-Ga_2_O_3_ photo-synapses with low weight update nonlinearity of 0.42 were developed by enhancing the concentration of self-trapped holes, thereby achieving multi-level in-sensor computing tasks. Theoretical and experimental investigations revealed that the interaction between the larger effective mass of holes and local lattice distortions in β-Ga_2_O_3_ promotes the formation of self-trapped holes, which significantly reduces hole mobility and enhances PPC effect. β-Ga_2_O_3_ films with a high concentration of self-trapped holes were prepared by increasing sputtering power from 60 W to 120 W, resulting in reduced hole mobility and a sustained high level of hole surface potential after 20 minutes decay, as revealed by KPFM measurements. The fabricated β-Ga_2_O_3_ photo-synapses demonstrated synaptic behaviors, including short-term plasticity with rapid decay time of 0.34 s and a slow decay time of 6.43 s, respectively, as well as the ability to transition from paired pulse facilitation to spike rate-dependent plasticity. By regulating light intensity, illumination duration, and frequency of 252 nm ultraviolet light, STP can be transformed into LTP, effectively simulating the learning-experience behavior of human brain. Meanwhile, the response peak and detective speed of the β-Ga_2_O_3_ photo-synapse remained unaffected. In practical applications, β-Ga_2_O_3_ photo-synapses achieved remarkable performance in image classification, anti-noise target tracking, and motion recognition, to validate its capability in in-sensor computing. For the image classification, the testing accuracy reached 99.48% on the MNIST dataset and 92.70% on the Fashion-MNIST dataset, closely matching the performance of commercial GPUs. In target tracking, the accuracy remained at 100% even under 60% noise intensity. Furthermore, a physical reservoir computing system based on a 2 × 8 β-Ga_2_O_3_ photo-synapses array achieved a recognition accuracy of 94.94% for 10 actions in UTD-MHAD dataset, approaching the 97.47% accuracy of commercial GPUs. This work highlights significant potential of β-Ga_2_O_3_ photo-synapses in neuromorphic in-sensor computing systems via self-trapped holes engineering, emphasizing the advantages in simple fabrication, low weight update nonlinearity, and suitability for industrial-scale applications. Our findings pave the way for the development of advanced neuromorphic devices with enhanced performance and practicality.

## Materials and methods

### Film growth and device fabrication

The gallium oxide thin films were deposited on c-plane sapphire substrates by radio frequency (RF) magnetron sputtering. During deposition, the flow rate of O_2_ and Ar kept at 4 SCCM and 40 SCCM, while sputtering pressure held on 1.4 Pa. To enhance the atomic peening effect, sputtering power increased from 60 W to 120 W. Then, the films were annealed at 800 °C in the air to convert to β-Ga_2_O_3_. Ti/Au (100 nm/300 nm) bilayer metal was deposited via magnetron sputtering as the electrodes upon the optimized β-Ga_2_O_3_ films, finished by the conventional photolithography and lift-off processes.

### Film characterizations and device characterizations

The crystal structure and quality of β-Ga_2_O_3_ films were investigated by X-ray diffraction (XRD, Rigaku D/max-2600/PC) using Cu Kα radiation (*λ* = 0.15418 nm). The distribution of elements in β-Ga_2_O_3_ films were analyzed by scanning electron microscope (SEM, Hitachi, SU70) with an attached Energy Dispersive Spectrometer (EDS). The composition of the films were obtained by X-ray photoelectron spectroscopy (XPS, ThermoFisher, ESCLAB 250Xi). The photoelectric test system, comprising a Xe lamp, a monochromator, an optical chopper, and the FS-pro380 semiconductor analyzer, was used to measure the synaptic behavior of the devices (*I*-*V* and *I*-*t* characteristics). By integrating β-Ga_2_O_3_ photo-synapses with non-volatile memristors, the solar-blind ultraviolet neuromorphic machine vision system was developed. Furthermore, the implementation of convolutional neural network and reservoir computing algorithms, along with the validation of system performance, was carried out by using Python program.

### DFT calculations

Our calculations are based on Vienna ab-initio simulation package (VASP). For gallium, the 4*s*, and 4*p* electrons are selected as valence electrons while the 2*s* and 2*p* electrons for oxygen. For the defect calculations, the HSE06 screen functional has been employed and the amount of exact exchange was set to 35%. A 120-atom (1× 3 × 2) supercell based on the 20-atom conventional unit cell of β-Ga_2_O_3_ was used. The cut-off energy for basis functions was 400 eV and atomic positions were relaxed until all the forces on atoms were below 0.01 eV Å^−1^. The presence of a uniform background charge was assumed to compensate the charged defects in the lattice and spin polarization was explicitly taken into account for defects with unpaired electrons.

## Supplementary information

Movie S1

Supplementary Information

## Data Availability

The data that support the findings of this study are available from the corresponding author upon reasonable request.
