# Supplementary material for: Two-terminal β-Ga2O3 photo-synapse for diversified in-sensor computing via self-trapped holes engineering
Source: Light Sci Appl. 2026 Jun 29;15:296. doi: 10.1038/s41377-026-02298-2 (PMC13315735; doi:10.1038/s41377-026-02298-2)
Supplement: Supplementary file 2 — Supplementary Information [file 41377_2026_2298_MOESM2_ESM.docx]

**Supplementary Information for**

Two-terminal β-Ga_2_O_3_ photo-synapse for diversified in-sensor computing via self-trapped holes engineering

Yiyin Nie,^1,2^ Shujie Jiao,^1,*^ Xing Yang,^2,3,**^ Shiyong Gao,^1^ Dongbo Wang,^1^ Yongfeng Li,^4^ Jinzhong Wang,^1^ Liancheng Zhao^1^

^1^School of Materials Science and Engineering, Harbin Institute of Technology, Harbin, 150001, China

^2^Advanced Laser Technology Laboratory of Anhui Province, Electronic Engineering Institute, National University of Defense Technology, Hefei, 230037, China

^3^Jianghuai Advance Technology Center, Hefei, 230037, China

^4^College of Physics, Jilin University, Changchun, 130012, China

*Corresponding author E-mail: [shujiejiao@hit.edu.cn](mailto:(shujiejiao@hit.edu.cn)); [yangxing17@nudt.edu.cn](mailto:(yangxing17@nudt.edu.cn))

**Sentence S1**

For β-Ga_2_O_3_, the presence of abundant oxygen vacancies renders the material highly susceptible to the chemical adsorption of ambient molecules such as H_2_O and CO_2_. These vacancies can spontaneously decrease in concentration by capturing O atoms from the surrounding environment, the process known as photocatalytic activity that is markedly accelerated under illumination. Previous studies have demonstrated that oxygen vacancy-rich β-Ga_2_O_3_ can photocatalytically convert atmospheric CO_2_ into carbonates or bicarbonates under ambient temperature and pressure conditions^1-3^. Concurrently, it can drive the oxygen evolution reaction (OER) to decompose H_2_O, yielding H_2_ and O_2_^4^. These reactions utilize CO_2_ and H_2_O as reactants and are sustained under light irradiation. Consequently, in illuminated environments, β-Ga_2_O_3_ photo-synapses with high concentration of oxygen vacancies are prone to undergo such photocatalytic processes, leading to disturbances in the stability of photocurrent. Furthermore, previous studies have also confirmed that variations in the concentration of oxygen vacancies in β-Ga_2_O_3_ can alter the interfacial contact properties between the electrode and the semiconductor, resulting in irreversible modifications to the *I-V* characteristics after photocatalytic processes^5,6^. Given that photo-synapse computing fundamentally relies on precise and controllable conductance, photocatalytically induced conductance-fluctuations may induce substantial computational errors, thereby adversely affecting computational outputs. Thus, although β-Ga_2_O_3_ photo-synapses based on oxygen vacancy strategy exhibit promising functionality, the long-term stability and reliability are significantly limited in practical applications due to the inherently photocatalytic reactivity with environmental H_2_O and CO_2_ under light exposure.

**Sentence S2**

For β-Ga_2_O_3_, the formation of self-trapped holes (STHs) in β-Ga_2_O_3_ arises from two factors. Firstly, the relatively flat valence band maximum (VBM) leads to the large hole effective mass, which constitutes a precondition for forming STHs. Secondly, the localization of holes as STHs occurs only in the presence of local lattice distortions. Therefore, STHs in β-Ga_2_O_3_ result from the synergistic interaction between relatively flat VBM and local lattice distortions, neither factor alone is sufficient to induce STHs^7,8^.

**Sentence S3**

The effective mass of electrons and holes can be calculated as Equation S1^9^:

$\frac{\text{1}}{m^{*}}\text{=}\frac{\text{1}}{\text{ℏ}^{\text{2}}}\frac{\text{∂}^{\text{2}}\text{E}}{{\text{∂}\text{k}}^{\text{2}}}$ **(Equation S1)**

Where $m^{*}$ is the effective mass of electrons ($m_{e}^{*}$) or holes ($m_{h}^{*}$), $\text{ℏ}$ is reduced Planck constant and *E* is the energy of the eigenstate referring to reciprocal *k* points. The calculated effective masses of electrons are 0.285 *m*_0_ (Γ - X|X_1_) and 0.284 *m*_0_ (Γ - Z), depending on the directions, and agreed well with 0.28 *m*_0_ tested in the experiments^10,11^. The calculated effective mass of holes ($m_{h}^{*}$) was 40 *m*_0_, owing to the relatively flat VBM of β-Ga_2_O_3_^10^.

**Sentence S4**

For deep-level defects that emit luminescence in PL spectrum, the relative concentrations of the i-th and j-th defects can be calculated by using Equation S2, providing that either the electron capture coefficient or the hole capture coefficient is known^12^:

$\frac{\text{N}_{\text{i}}}{\text{N}_{\text{j}}}\text{=}\frac{\text{C}_{\text{j}}}{\text{C}_{\text{i}}}\frac{\text{I}_{\text{i}}^{\text{PL}}}{\text{I}_{\text{j}}^{\text{PL}}}$ **(Equation S2)**

Where $\text{N}_{\text{i}}$ and $\text{N}_{\text{j}}$ are the concentrations of i-th and j-th defects, $\text{C}_{\text{i}}$ and $\text{C}_{\text{j}}$ are the electron capture coefficient or the hole capture coefficient of i-th and j-th defects, $\text{I}_{\text{i}}^{\text{PL}}$ and $\text{I}_{\text{j}}^{\text{PL}}$ are the luminescence intensity of i-th and j-th defects.

Based on Equation S2, the relative percentage of the i-th defect among the total luminescent defects can be calculated by using Equation S3:

$\text{α}_{\text{N}_{\text{i}}}\text{=}\frac{\text{C}_{\text{i}}\text{I}_{\text{i}}^{\text{PL}}}{\sum_{\text{j}} \text{C}_{\text{j}}\text{I}_{\text{j}}^{\text{PL}}}\text{×100 \%}$ **(Equation S3)**

Where $\text{α}_{\text{N}_{\text{i}}}$ is the percentage of the i-th defect in the total luminescence defects. When the types of luminescent defects are fixed, the concentration variation of the i-th defect can be inferred from the changed percentage of its intensity relative to the total intensity of all luminescent defects.

**Sentence S5**

For XRD pattern, the grain size of β-Ga_2_O_3_ films can be calculated through the Scherrer formula. However, STHs in β-Ga_2_O_3_ films originated from local lattice distortions induced by the atomic peening effect during sputtering, instead of being caused by the variation of grain size. As the sputtering power increased, the grain size of the β-Ga_2_O_3_ films gradually increased, which was reflected in the XRD patterns as a gradual narrowing of the FWHM of $\text{(}\bar{\text{2}}\text{01)}$ diffraction peaks. If the STHs were mainly caused by stress distortion of the grain sizes, β-Ga_2_O_3_ films grown under 60 W sputtering power with the smallest grain size should have the highest concentration of STHs. However, the results had shown that β-Ga_2_O_3_ films with the highest concentration of STHs corresponded to the sputtering power of 120 W. Thus, there was no obvious correlation between the STHs and the grain size.

**Sentence S6**

In this work, the PPC effect and synaptic behavior of β-Ga_2_O_3_ photo-synapses are mainly attributed to STHs, this conclusion is supported by the following three points:

Firstly, photo-synapses based on the oxygen vacancy strategy involve ionization and migration of oxygen vacancies under external light stimulation. Due to the inherently slow kinetics of ion migration, the rising time of *I*-*t* curves is relatively long. In contrast, photo-synapses based on the STHs strategy involve only hole localization upon light excitation, resulting in a significantly faster rising time. The distinct *I*-*t* characteristics arising from these two mechanisms are thoroughly discussed in the Fig. S1 of Supplementary Information.

Secondly, XPS and PL analyses have demonstrated that the concentration of oxygen vacancies in the β-Ga_2_O_3_ films did not change significantly, whereas increasing the sputtering power selectively enhanced the concentration of STHs. Consequently, in films prepared at 60 W and 120 W, only the concentration of STHs varied, while the concentration of oxygen vacancies remained nearly unchanged. Under these conditions, the sample fabricated with 60 W sputtering power exhibited no PPC effect and showed a very fast response speed, whereas the sample prepared at 120 W displayed a pronounced PPC effect. This indicated that the trace amount of oxygen vacancies in our β-Ga_2_O_3_ films was insufficient to induce PPC effect, and the observed PPC effect was mainly attributable to the increased concentration of STHs achieved at higher sputtering power.

Finally, the variation in electron and hole decay times in films prepared at 60 W and 120 W sputtering powers was directly observed using KPFM. It is revealed that the hole decay time in the film fabricated at 120 W was significantly prolonged, which was consistent with the trapped-detrapped mechanism and hopping dynamics associated with STHs. In contrast, the electron decay time remained nearly unchanged across samples prepared under different sputtering powers, a behavior that contrasted sharply with the characteristic signature of *V*_O_-based photo-synapses, in which the PPC effect originated from extended electron decay. Based on the above evidence, it could be concluded that PPC effect and synaptic behavior of β-Ga_2_O_3_ photo-synapses in this work are mainly attributed to STHs.

**Sentence S7**

Generally, weights in the neural network are floating-point numbers that can be positive or negative. However, the weights of our β-Ga_2_O_3_ synapses are positive. To address the limitation that physical synapses only exhibit positive conductance, we implemented a differential pair architecture where each synaptic weight is mathematically represented by the conductance difference between two parallel devices (an excitatory device and an inhibitory device). This approach effectively maps the strictly positive physical conductance values to the signed (positive and negative) weight space required by the neural network, allowing the system to represent negative weights without requiring negative physical conductance.

Besides, the conductance of the actual synapse is small, which would lead to the explosion or disappearance of gradients in CNN. Regarding the issues of small conductance values and gradient stability, we adopted a Quantization-Aware Training (QAT) framework combined with a Straight-Through Estimator (STE). Instead of directly using the minuscule physical conductance values for backpropagation, we normalized the physical states to a standard weight range and used the STE algorithm to approximate gradients during the backward pass. This strategy effectively bypasses the non-differentiable nature of discrete physical states and prevents gradient disappearance or explosion, ensuring robust network convergence despite the hardware constraints.

The distinction between the photo-synapse simulation and the 4060 GPU implementation lies in the precision of the weights. The 4060 GPU results represent an ideal software baseline using standard high-precision floating-point weights (Float32), whereas the photo-synapse simulation represents a hardware-constrained model where weights are strictly limited to the discrete, non-uniform quantization levels physically achievable by our devices. The results in Fig. 5f-i demonstrate that the accuracy of the quantized simulation closely matches the ideal GPU baseline, confirming that the finite precision of our photo-synapses is sufficient for high-performance image classification tasks.

**Sentence S8**

For β-Ga_2_O_3_ photo-synapses, the decay process of synaptic devices inherently induces a temporal dimension, creating a spatiotemporal motion tailing effect in practical scenarios. For the Object Tracking task, the motion tailing is a secondary background signal, whereas the peak response at the target’s current position is the dominant decision-making factor. Therefore, simplifying the visual representation to focus on the dominant signal allows for a clearer explanation of the working principle without altering the tracking conclusion.

To theoretically justify why the tracking result remains robust and unchanged even if the motion tailing is fully rendered, we model the total current response $\text{I}_{\text{total}}$ as the superposition of the instantaneous optical stimulus $\text{I}_{\text{stim}}$ and the decaying residual from previous frames:

$\text{I}_{\text{total}}\left( \text{x}\text{,}\text{t} \right)\text{=}\text{I}_{\text{stim}}\left( \text{x}\text{,}\text{t} \right)\text{+}\sum_{\text{k}\text{=1}}^{\text{N}} \text{I}_{\text{total}}\left( \text{x}\text{,}\text{t}\text{−}\text{k} \right)\text{·}\text{e}^{\text{−}\text{k}/\text{τ}}$ **(Equation S4)**

where $\text{I}_{\text{total}}\left( \text{x}\text{,}\text{t} \right)$ represents the total current response at position $\text{x}$ and time $\text{t}$, and $\text{I}_{\text{stim}}\left( \text{x}\text{,}\text{t} \right)$ denotes the instantaneous photocurrent generated by the optical stimulus (the target). The summation term represents the motion tailing from previous frames, where $\text{k}$ is the time lag, $\text{N}$ is the effective memory length, and $\text{τ}$ is the decay time constant of the device.

The tracking algorithm calculates the Normalized Cross-Correlation between the stored high-fidelity template *T* and the real-time frame:

$\text{R}\left( \text{u}\text{,}\text{v} \right)\text{=}\frac{\sum_{\text{x}\text{,}\text{y}} \left[ \text{I}_{\text{total}}\left( \text{x}\text{+}\text{u}\text{,}\text{y}\text{+}\text{v}\text{,}\text{t} \right)\text{·}\text{T}\left( \text{x}\text{,}\text{y} \right) \right]}{\sqrt{\sum\text{I}_{\text{total}}^{\text{2}}\text{·}\sum\text{T}^{\text{2}}}}$ **(Equation S5)**

In the formula, $\text{R}\left( \text{u}\text{,}\text{v} \right)$ is the Normalized Cross-Correlation coefficient at shift $\left( \text{u}\text{,}\text{v} \right)$. $\text{T}\left( \text{x}\text{,}\text{y} \right)$ represents the stored high-fidelity template of the target. $\sum\text{I}_{\text{total}}^{\text{2}}$ and $\sum\text{T}^{\text{2}}$ are the normalization factors corresponding to the energy of the real-time frame patch and the template, respectively.

Since the template *T* corresponds to the distinct features of the target, its correlation with the instantaneous signal is significantly stronger than its correlation with the decaying tail. Mathematically:

$\text{R}\left( \text{Target} \right)\text{≈1.0}\text{≫}\text{R}\left( \text{Tail} \right)\text{≈α}\text{·}\text{R}\left( \text{Target} \right)$ **(Equation S6)**

where $\text{α}$ represents the attenuation factor of the motion tail ($\text{α}$ < 1), demonstrating that the correlation score of the motion tail ($\text{R}\left( \text{Tail} \right)$) is significantly lower than that of the target $\text{R}\left( \text{Target} \right)$, allowing for effective threshold-based filtering.

Consequently, the simulation in Fig. 6 effectively captures the governing physics required for accurate tracking. Including the motion tailing effect would increase the computational complexity of the model but would not alter the final identification of the maximum likelihood position.

**Sentence S9**

Specifically, the tracking accuracy ($\text{η}$) is defined as the percentage of video frames where the system successfully retrieves the target from the background. A frame is considered successfully tracked if the normalized cross-correlation score ($\text{R}$). between the device-stored template and the current frame exceeds a valid detection threshold ($\text{θ}$). Mathematically:

$\text{η}\text{=}\frac{\text{N}_{\text{success}}}{\text{N}_{\text{total}}}\text{×100\%}$ **(Equation S7)**

$\text{N}_{\text{success}}\text{=}\sum_{\text{t}\text{=1}}^{\text{N}_{\text{total}}} \text{I}\left( \text{R}_{\text{t}}\text{>}\text{θ} \right)$ **(Equation S8)**

where $\text{N}_{\text{total}}$ is the total number of frames (77 frames), $\text{I}$ is the indicator function. This definition treats tracking under high noise as a signal retrieval problem. As shown in Fig. 6d, this metric effectively quantifies the drop-off point where the noise intensity exceeds the device’s memory retention capability, providing a clear indicator of the hardware’s physical limit.

**Sentence S10**

The UTD-MHAD dataset contains 27 classes of human actions collected from 8 experimenters. Each skeleton frame consists of dynamic coordinates of 20 body joints. In this article, we only selected 10 different actions for training, which are “right arm swipe to the left (swipe_left)”, “right hand wave (wave)”, “hand throw (throw)”, “basketball shooting (basketball)”, “front boxing (boxing)”, “tennis forehand swing (tennis)”, “jogging (jogging)”, “sit to stand (sit to stand)”, “forward lunge (lunge)” and “squat (squat)”, respectively.

The data flow based on the RC architecture utilizing virtual nodes via time-multiplexing are processed as follows:

1. 3D Signal Transformation:

The human skeleton data is aggregated into 5 body groups (torso, left/right arm, left/right leg). For each group, the 3D spatial coordinates are extracted. This results in 15 parallel feature streams, which are modulated into optical pulses to stimulate the 15 physical synapses.

2. Expansion from 15 Currents to 720 Features:

To map these low-dimensional inputs to a high-dimensional feature space, we utilize the temporal virtual node concept inherent to reservoir computing. Leveraging the fading memory and non-linear dynamics of the devices, we employ a time-multiplexing technique. For each of the 15 physical devices, the output photocurrent response is sampled at 48 distinct time steps (virtual nodes) within the processing window.

3. Current-to-Voltage Conversion:

At the device interface, the characteristic photocurrents from the 15 devices are converted into voltage signals via sampling circuits. These 720 voltage values are then mapped to the word lines of the non-volatile memristor array (readout layer) , where the matrix multiplication is performed physically to classify the 10 actions.

In the simulation framework, this physical VMM process is mathematically modeled using Ridge Regression. The training phase calculates the optimal output weights that minimize the classification error, and these calculated weights conceptually correspond to the target conductance values that would be programmed into the physical NVM array. This allows us to accurately evaluate the system’s theoretical performance limits using experimentally calibrated device parameters.

The current work represents a device-level validation and system-level simulation based on physical characteristics, rather than a fully integrated hardware prototype. However, the successful verification of the sensing-computing mechanism and the robust classification results obtained under physical constraints strongly validate the feasibility of this architecture. This study, therefore, establishes a solid foundation and provides a clear technological roadmap for the future development of fully hardware-implemented, all-optical in-sensor reservoir computing systems.

**Sentence S11**

Ridge Regression is indeed widely employed for multi-class classification in reservoir computing frameworks through one-hot encoding of class labels, which is a standard and theoretically well-established approach in the field. Specifically, for our 10-class motion recognition task, each training label is encoded as a one-hot vector $\text{y}_{\text{i}}\text{∈}\{\text{0,1}{\}}^{\text{10}}$ where only one element equals 1 and the others are 0. Ridge Regression then learns a weight matrix $\text{W}\text{∈}\text{R}^{\text{10}\text{×}\text{720}}$ by minimizing the regularized loss function $\text{ℒ=}|\text{Y−WX}|_{\text{F}}^{\text{2}}\text{+α}|\text{W}|_{\text{F}}^{\text{2}}$, where $\text{X}$ represents the 720-dimensional reservoir states and $\text{α}$ is the regularization parameter. Classification is then performed by computing the output vector $\text{o}\text{=}\text{Wx}$, for a test sample and selecting the class with maximum value: $\hat{\text{y}}\text{=}\arg\max_{\text{k}} \text{o}_{\text{k}}$. This formulation is mathematically equivalent to training k independent regression models simultaneously, one for each class, and has been extensively validated in reservoir computing literature, including seminal works by Mantas Lukoševičius et al. and Gouhei Tanaka et al.^45,46^

**Sentence S12**

Fig. 7b and Fig. S28 collectively demonstrate the complete physical encoding basis that enables our β-Ga_2_O_3_ photo-synapses to function as a physical reservoir layer. Specifically, Fig. 7b presents the photocurrent decay curves for four representative 4-bit encoding patterns (“1000”, “1001”, “1010”, “1100”), while Fig. S28 provides the remaining twelve patterns, together constituting all sixteen possible 4-bit combinations (2^4^ = 16 encoding states from “0000” to “1111”). These sixteen distinct temporal response patterns form the physical computational basis of the reservoir layer, this is not merely a characterization dataset, but rather the fundamental mechanism through which the devices perform physical computation.

In our reservoir computing architecture, the human motion coordinates from 5 body groups are encoded into these 4-bit optical pulse patterns, and each of the 15 physical photo-synapses transforms its input through the device's intrinsic volatile dynamics (characterized by photocarrier generation, trapping, and recombination processes) to generate time-dependent photocurrent responses. The complete set of sixteen encoding patterns demonstrates that our devices can reliably map different input combinations into distinguishable temporal signatures, which is essential for the reservoir’s computational expressiveness. The value of this physical encoding scheme lies in its implementation of in-material computing: the non-linear transformation and temporal integration required for reservoir computing are performed directly by the physics of the β-Ga_2_O_3_ material rather than through digital computation or explicit programming. When combined with the time-multiplexing strategy, where each device’s response is sampled at 48 discrete time points, these sixteen physical encoding states enable each device to generate 48 virtual nodes with distinct temporal characteristics. Across 15 devices, this creates 15 × 48 = 720 high-dimensional features that collectively encode multi-scale spatiotemporal information about the motion trajectory. The sixteen patterns shown in Fig. 7b and Fig. S28 are therefore not simply device characterization curves, but rather constitute the physical reservoir kernel that maps low-dimensional motion inputs into a high-dimensional feature space suitable for linear classification. This approach has been widely reported in previous literature, such as the recent work of Huang et al. on ZnO memristors^47^.


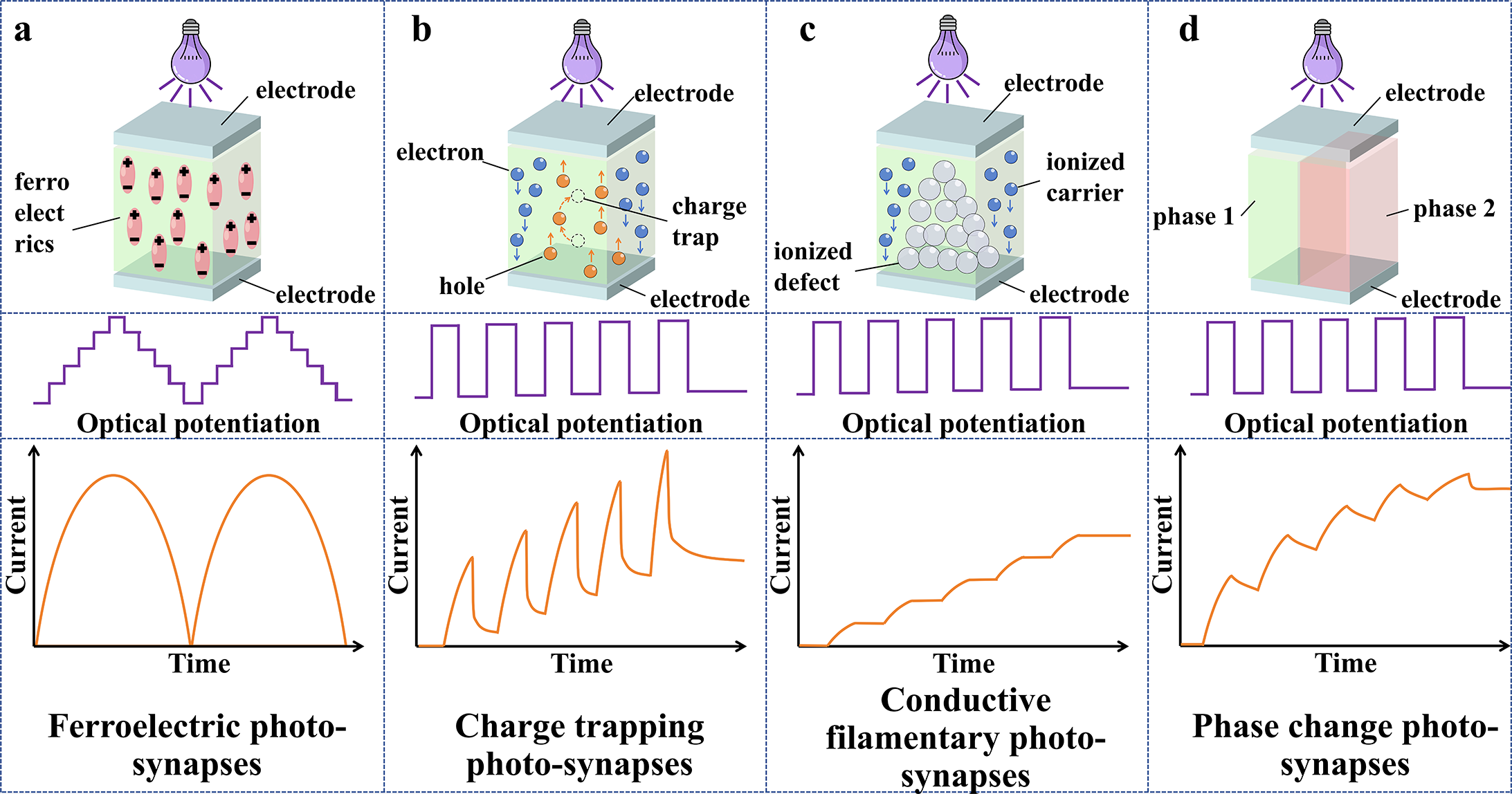


**Fig. S1** **The physical mechanisms and *I*-*t* curves underlying different types of photo-synapses. a** Ferroelectric photo-synapses. **b** Charge trapping photo-synapses. **c** Conductive filamentary photo-synapses. **d** Phase change photo-synapses.

The responsivity of ferroelectric photo-synapses is controlled by adjusting the barrier height and built-in electric field through ferroelectric polarization, as illustrated in Fig. S1a. This enables both positive and negative responses and performs in-situ matrix-vector multiplication between input images and responsivity matrices.

Charge trapping photo-synapses in Fig. S1b modulate conductivity by capturing photogenerated carriers at defects such as STHs. These devices offer a simple fabrication process and rapid photoresponse characteristics. However, the carrier trapping process is inherently dynamic and transient, leading to volatile conductance changes that are incompatible with long-term data retention. As a result, charge trapping photo-synapses are well suited for in-sensor computing, especially in time-dependent tasks.

Conductive filamentary photo-synapses in Fig. S1c modulate conductivity via ionization defects such as oxygen vacancies. Under illumination, oxygen vacancies ionize and migrate to the cathode to form conductive filaments, while electrons move to the anode. These devices are non-volatile and well suited for in-memory computing. However, slow migration of ionization defects limits response speed, making them unsuitable for in-sensor computing.

Phase change photo-synapses in Fig. S1d control conductivity via light-induced phase transitions, offering high multi-level storage capacity and non-volatility. However, high energy consumption limits their efficiency^13^.


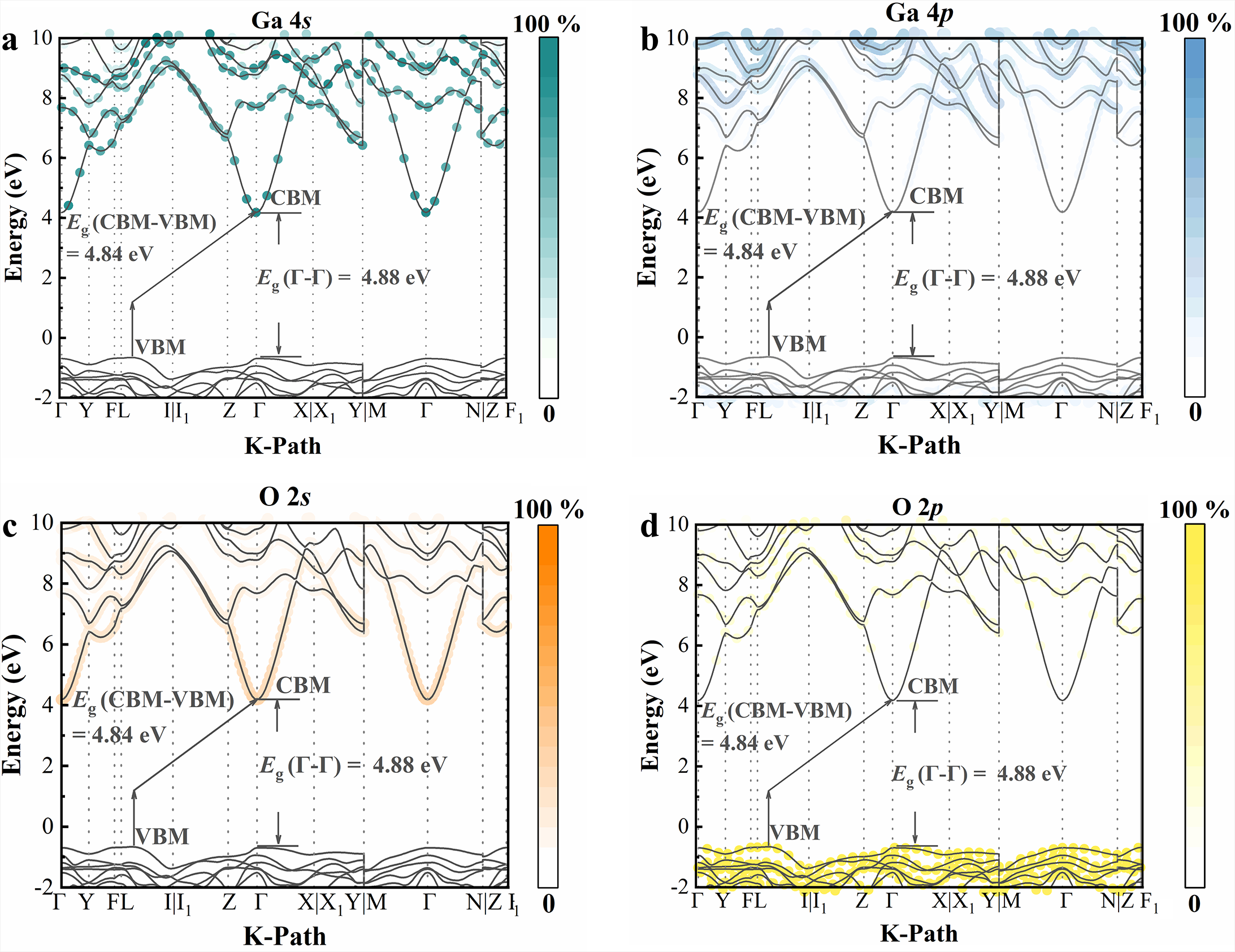


**Fig. S2** **Projected band structure of β-Ga_2_O_3_.** **a** Ga 4*s* states. **b** Ga 4*p* states. **c** O 2*s* states. **d** O 2*p* states.


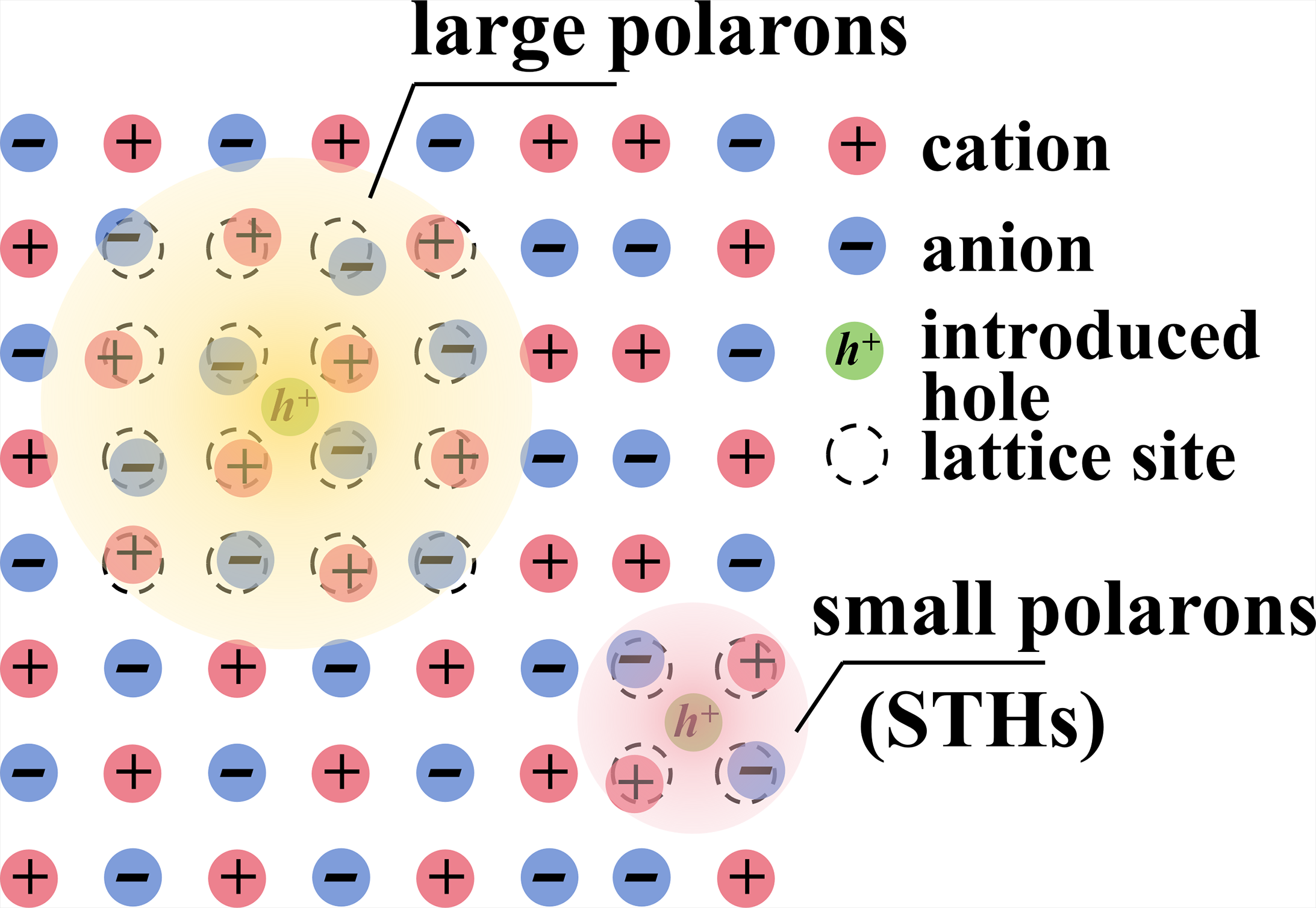


**Fig. S3** **Mechanism of large polarons and small polarons.**

Polarons characterized by a distortion exceeding lattice constant are termed large polarons, resulting in a further increase in effective mass of carriers. In contrast, polarons confined within a single unit cell are referred to as small polarons, contributing to PPC effect by limiting carrier mobility.


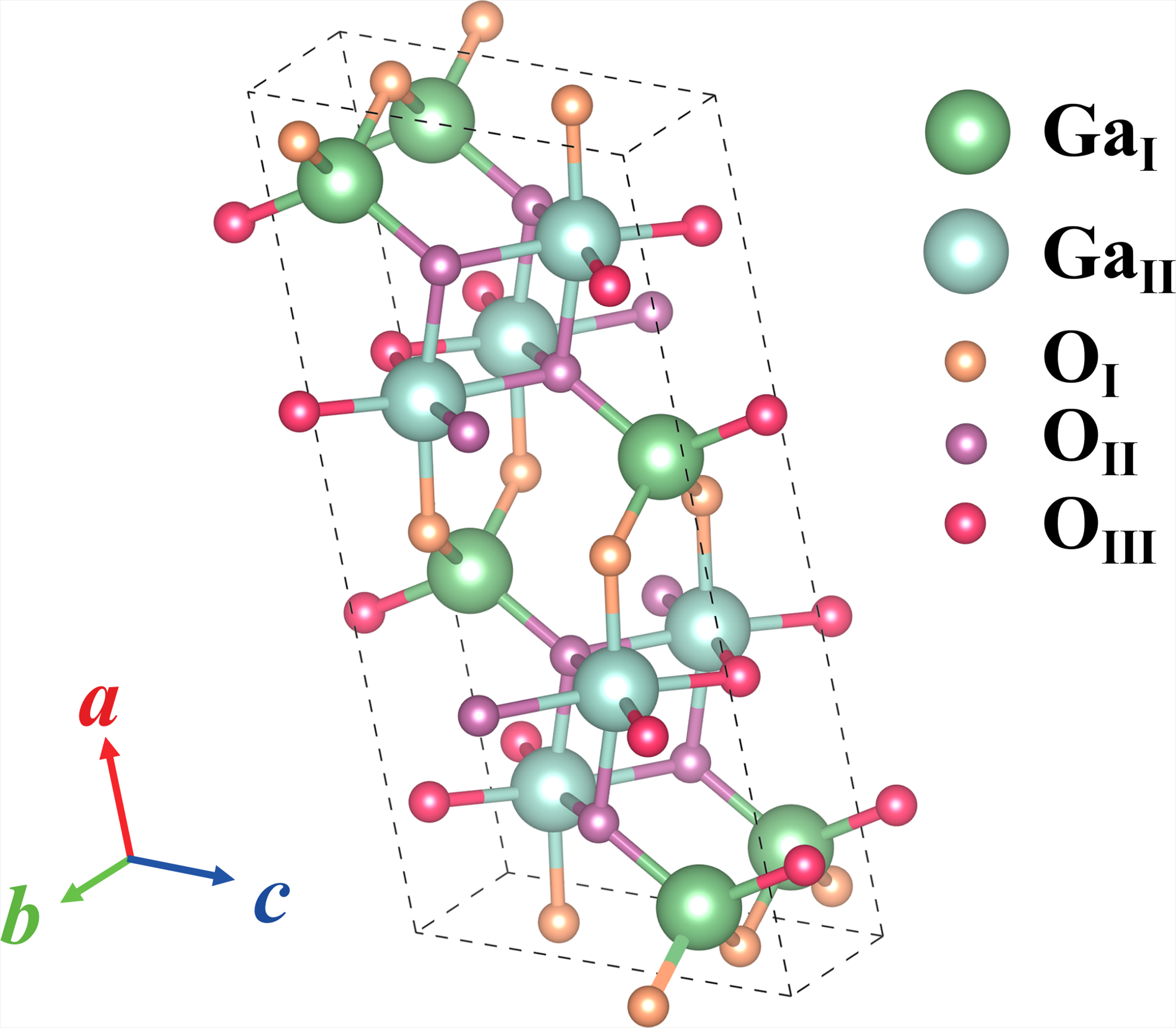


**Fig. S4** **Crystal of β-Ga_2_O_3_.**

β-Ga_2_O_3_ unit cell with two crystallographically nonequivalent gallium ions (Ga_Ⅰ_ and Ga_Ⅱ_) and three nonequivalent oxygen ions (O_Ⅰ_, O_Ⅱ_ and O_Ⅲ_) was illustrated in Fig. S4. The calculated structural parameters were *a* = 12.27 Å, *b* = 3.05 Å, *c* = 5.83 Å, *β* = 103.82 °, consistent with experimental measurements.


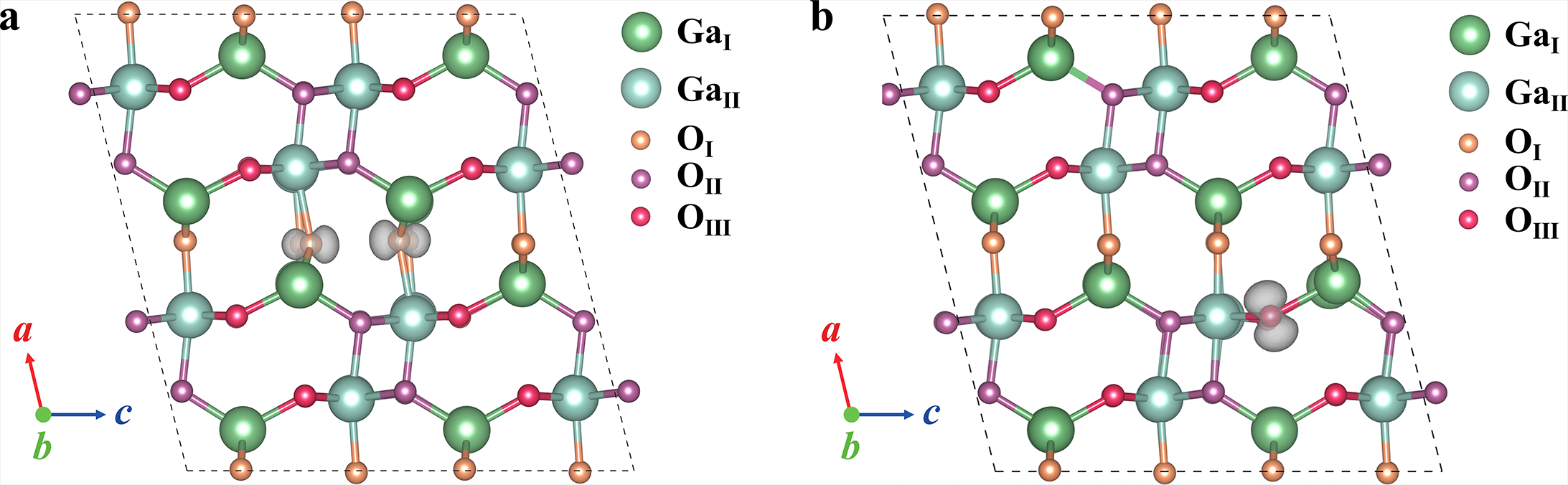


**Fig. S5 Charge distribution diagram of β-Ga_2_O_3_ with self-trapped holes. a** Self-trapped holes at O_Ⅰ_ atoms. **b** Self-trapped holes at O_Ⅲ_ atoms.


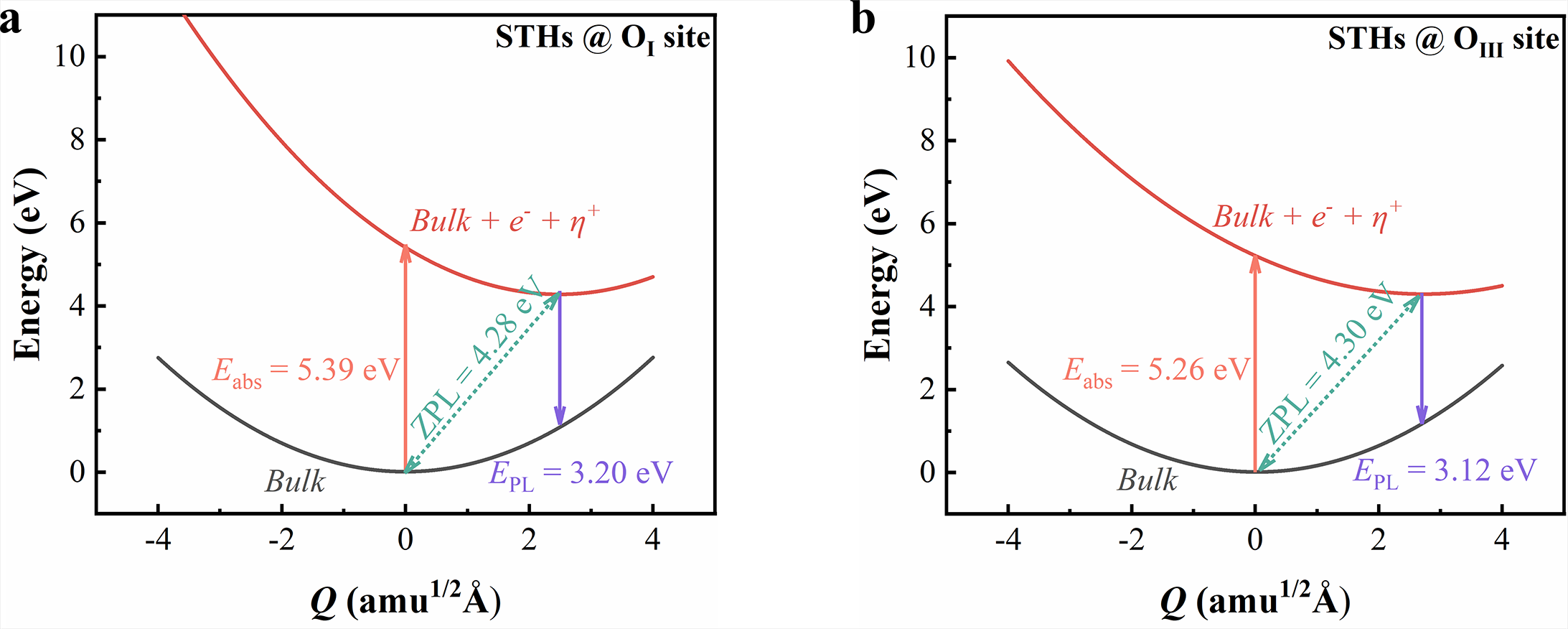


**Fig. S6 Configuration coordinate graph of self-trapped holes. a** Self-trapped holes at O_Ⅰ_ atoms. **b** Self-trapped holes at O_Ⅲ_ atoms.

As shown in Fig. S6, $\text{STHs}_{\text{@}\text{O}_{\text{Ⅰ}}}$ and $\text{STHs}_{\text{@}\text{O}_{\text{Ⅲ}}}$ exhibit (+/0) thermodynamic transition levels, indicating that STHs remain in 0 charge state at the ground state (the black curve). Upon ultraviolet irradiation of β-Ga_2_O_3_, photogenerated electrons at the valence band are excited to the conduction band, while the corresponding photogenerated holes in the valence band are captured by STHs. This process could result in $\text{STHs}^{\text{0}}$ converting to $\text{STHs}^{\text{+}}$ (*η*^+^ in Fig. S6) and the system remaining in the excited state. Due to the instability of the excited state, radiative recombination between the trapped holes in STHs and electrons occurs, resulting in the emission of ultraviolet light at energies of 3.20 eV ($\text{STHs}_{\text{@}\text{O}_{\text{Ⅰ}}}$) and 3.12 eV ($\text{STHs}_{\text{@}\text{O}_{\text{Ⅲ}}}$). This process is well described by the configuration coordinate graph derived from DFT calculations. Notably, the scenario in Fig. S6 corresponds to direct generation of photogenerated carriers followed by immediate hole-capture by STHs, which requires the absorption energy (*E*_abs_) exceeding the bandgap of β-Ga_2_O_3_. The more prevalent mechanism involves initial absorption of energy matching the bandgap followed by the migration of holes toward STH sites, where they are subsequently captured, as illustrated in Fig. 2c and 2f. Thus, the intensity of ultraviolet emission in the PL spectrum can serve as the reliable signal for monitoring changes in the concentration of STHs^14-18^.


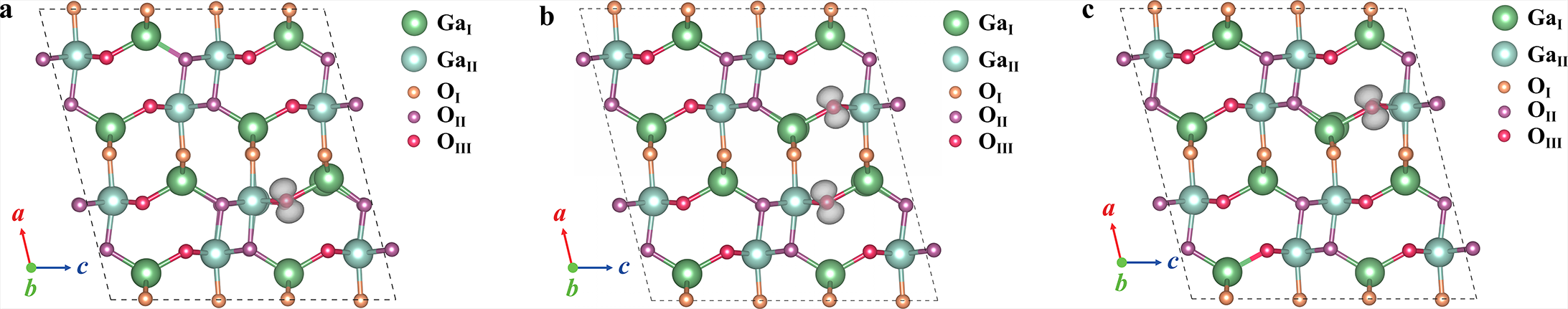


**Fig. S7 Charge distribution diagram of self-trapped holes at O_Ⅲ_ atoms under hopping mechanism. a** Initial configurations (*x* = 0). **b** Intermediate configurations (*x* = 0.5). **c** Final configurations (*x* = 1).

The trapped-detrapped process and the hopping mechanism represent intrinsic characteristics inherent to self-trapped holes (STHs). The unoccupied states of STHs enable the capture of holes from the valence band, resulting in a transition from $\text{STHs}^{\text{0}}$ to $\text{STHs}^{\text{+}}$. Also, $\text{STHs}^{\text{+}}$ can attract electrons to return to $\text{STHs}^{\text{0}}$, simultaneously releasing the localized holes back into the valence band. The trapped-detrapped process is particularly pronounced under non-equilibrium conditions such as illumination, as is the hopping mechanism. In hopping mechanism, small polarons are excited out of the self-trapping potential wells and migrate to adjacent lattice sites by transferring to another self-trapped state, accompanied by local lattice distortions, which can be described within the Marcus’ theory. Therefore, both the trapped-detrapped process and hopping mechanism must be considered when evaluating the impact of STHs on hole mobility in β-Ga_2_O_3_.

As shown in Fig. S7 and Fig. S8, a STH was excited out of its self-trapping potential well and subsequently hopped to another neighboring O_Ⅲ_ site with photo-assistant. The migration barrier ($\text{E}_{\text{b}}$) could be calculated using the coordinates of intermediate configurations, defined as $\text{R}\text{ }\text{=}\text{ }\text{x}\text{R}_{\text{i}}\text{+(1−}\text{x}\text{)}\text{R}_{\text{f}}$, where $\text{R}$, $\text{R}_{\text{i}}$ and $\text{R}_{\text{f}}$ represent the coordinates of all atoms in β-Ga_2_O_3_ associated with intermediate, initial and final configurations, $\text{x}$ is interpolation parameter for intermediate configuration. $\text{E}_{\text{b}}$ for a STH in O_Ⅲ_ sites was 516.6 meV and the related absorption energy could be estimated as $\text{E}_{\text{abs}}\text{ }\text{=}\text{ }\text{4}\text{E}_{\text{b}}$ = 2.07 eV^19^. The hopping mobility ($\text{μ}_{\text{hopping}}$) of STHs could be approximated as $\text{μ}_{\text{hopping}}\text{ }\text{=}\text{ }\text{[}{\text{e}\text{a}^{\text{2}}\text{ω}_{\text{0}}}/{\text{k}_{\text{B}}\text{T}}\text{][exp(}{\text{−}\text{E}_{\text{b}}}/{\text{k}_{\text{B}}\text{T}}\text{)]}$^19-21^, where $\text{a}\text{ }\text{=}$ 3.80 Å is the hopping distance and $\text{ω}_{\text{0}}$ is the longitudinal phonon frequency. The large $\text{E}_{\text{b}}$ would result in lower $\text{μ}_{\text{hopping}}$ at room temperature, which implies that once photogenerated holes would be captured by STHs, they can still migrate through hopping mechanisms. However, the overall mobility will be significantly reduced.





**Fig. S8 Migration energy barrier for hopping STHs on O_Ⅲ_ atoms.**


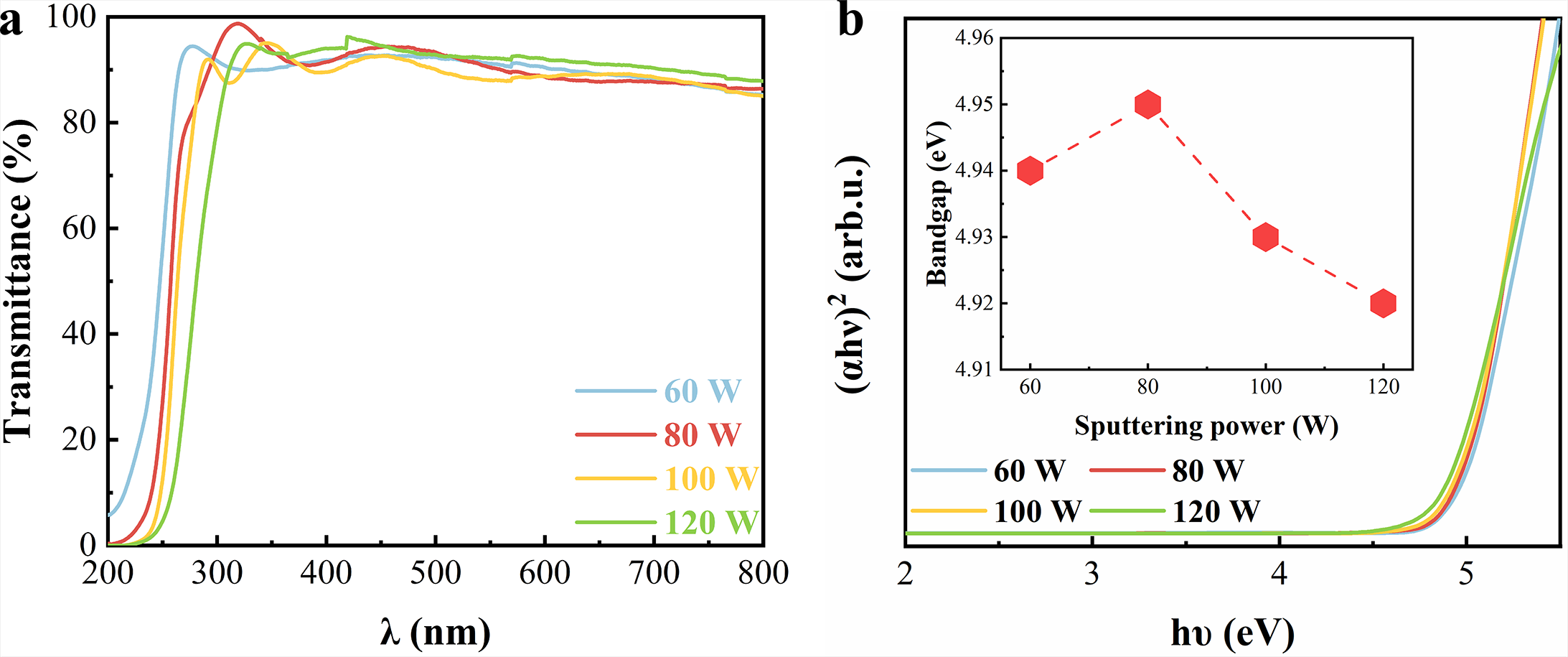


**Fig. S9** **Transmittance spectra of β-Ga_2_O_3_ films grown under various sputtering power.** **a** Transmittance. **b** Optical bandgap

In Fig. S9a, the evident red-shift in the transmission spectra of β-Ga_2_O_3_ films was observed with increasing sputtering power. This phenomenon could be attributed to the presence of a critical absorption thickness, *t*_α_, for incident light with photon energy exceeding the bandgap of β-Ga_2_O_3_. When the thickness is below *t*_α_, increasing thickness leads to enhanced light absorption, resulting in reduced transmittance and a corresponding red-shift in the spectrum. In contrast, once the film thickness exceeds *t*_α_, further increasing film thickness has almost no effect on optical absorption, and the transmission spectra become nearly identical across samples. Based on our previous researches, *t*_α_ of β-Ga_2_O_3_ is estimated to be approximately 400 nm^22,23^. Obviously, the thicknesses of β-Ga_2_O_3_ films in Fig. S10 were below this limit. Consequently, as the sputtering power is increased from 60 W to 120 W, the film thickness increases, leading to stronger light absorption and the red-shift in the transmission spectra.

To verify that the bandgap of β-Ga_2_O_3_ remains stable under various sputtering powers, the optical bandgap of β-Ga_2_O_3_ films deposited at various sputtering powers were calculated via Tauc-plot method in Fig. S9b, plotting (*αhν*)^2^ as the function of *hν*. The absorption coefficient α is evaluated by^22,23^:

$\alpha= -\frac{lnT}{t}$ **(Equation S9)**

Where *T* is the optical transmittance shown in Fig. S9a, *t* is the thickness of β-Ga_2_O_3_ films in Fig. S10. The optical bandgap of β-Ga_2_O_3_ films exhibited minor variation, ranging narrowly from 4.91 eV to 4.95 eV in Fig. S9b, thereby indicating that the observed spectral red-shift resulted from thickness-dependent absorption attenuation, rather than the modification of the intrinsic bandgap induced by sputtering power.


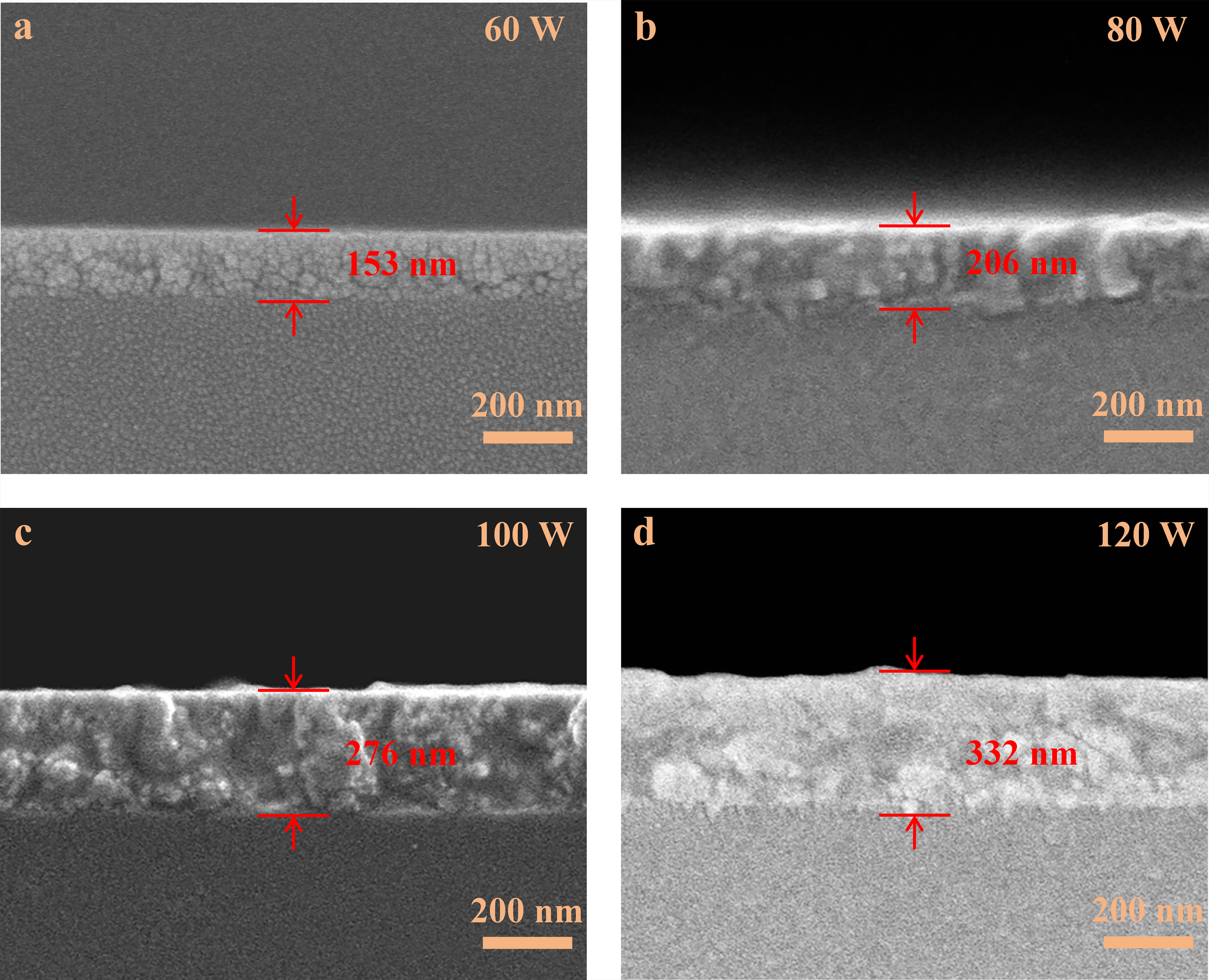


**Fig. S10** **Film thickness of β-Ga_2_O_3_ films grown under various sputtering powers. a** 60 W. **b** 80 W. **c** 100 W. **d** 120 W.

**Fig. S11 XRD of β-Ga_2_O_3_ films grown under various sputtering powers.**





**Fig. S12 XPS of β-Ga_2_O_3_ films grown under various sputtering powers.**


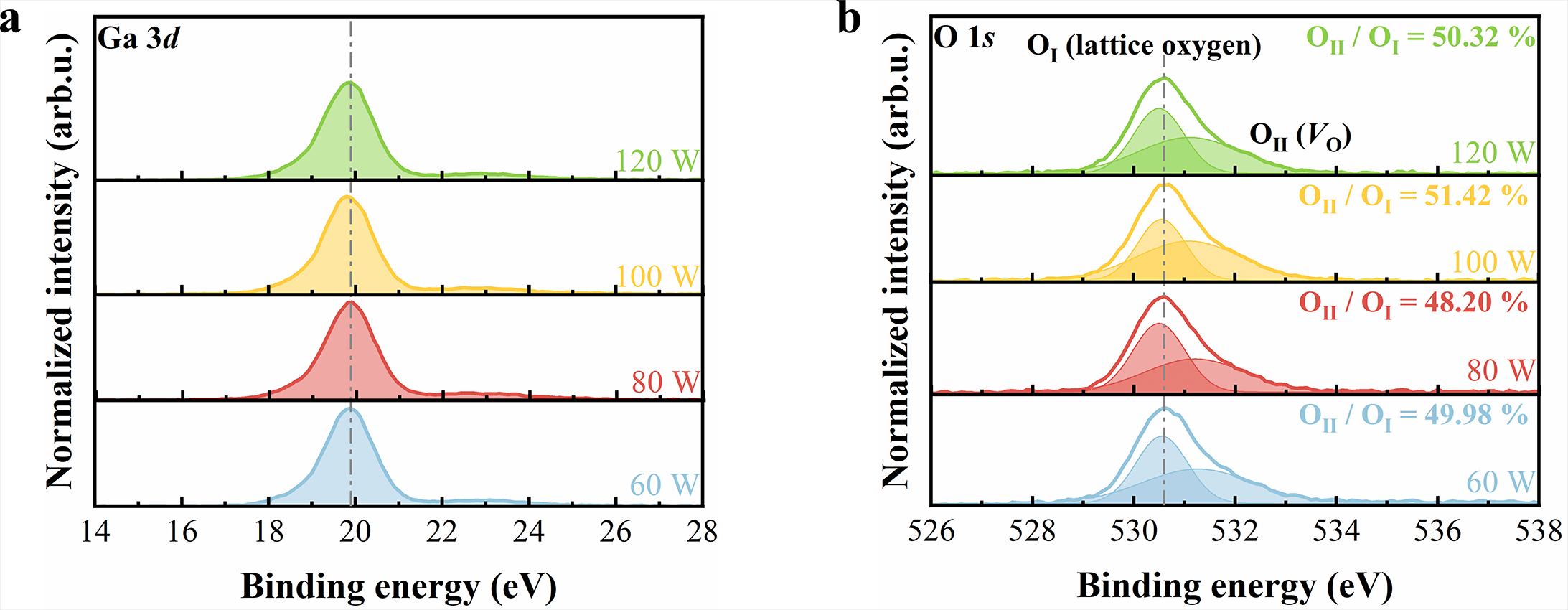


**Fig. S13 XPS of Ga and O elements in β-Ga_2_O_3_ films grown under various sputtering powers. a** Ga 3*d*. **b** O 1*s*.

O 1*s* peaks were divided into two separated peaks in Fig. S13. The peaks around 530.8 eV were signed as O_Ⅰ_, resulting from lattice oxygen. Other peaks around 532.4 eV were O_Ⅱ_, associating with oxygen vacancies (*V*_O_). With sputtering power increasing from 60 W to 120 W, the percentage of O_Ⅱ_ remained relatively consistent. The results of XPS indicated that increased sputtering power did not significantly modify growth environment of β-Ga_2_O_3_ films, which consequently had minimal impact on the concentration of defects associated with Ga and O atoms (*V*_Ga_, *V*_O_, Ga*_i_* and O*_i_*).


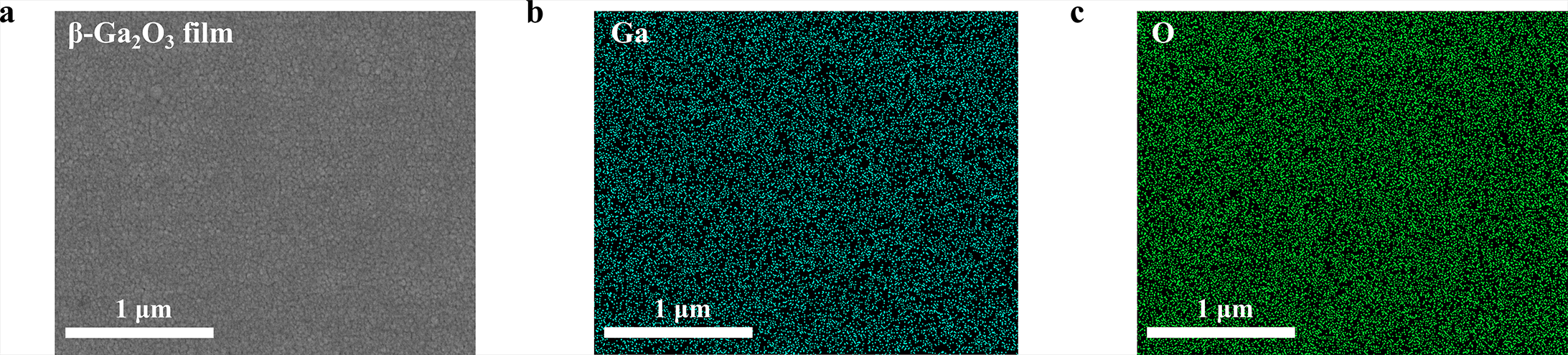


**Fig. S14 EDS mapping of β-Ga_2_O_3_ film grown under 120 W.**

As depicted in Fig. S14, Ga and O elements are uniformly distributed in β-Ga_2_O_3_ film grown under 120 W, suggesting the increase in concentration of STHs was not caused by element segregation.





**Fig. S15 PL spectrum of β-Ga_2_O_3_ films grown under various sputtering powers.**


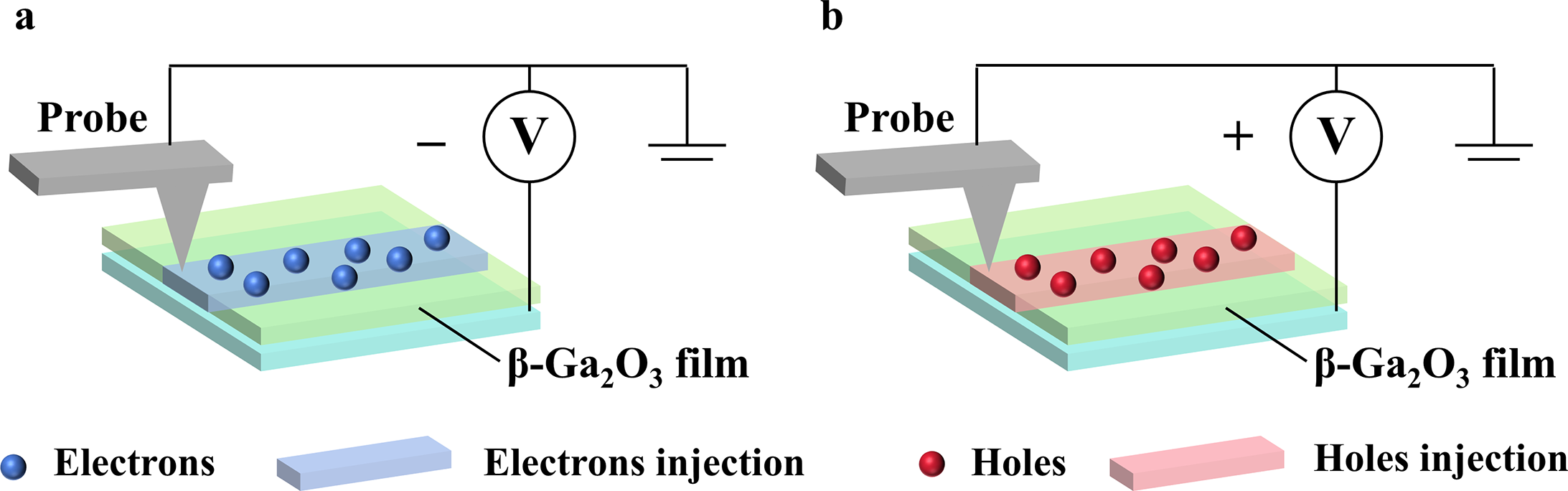


**Fig. S16 Schematic diagram of KPFM measurement on β-Ga_2_O_3_ films. a** Injecting electrons. **b** Injecting holes.

KPFM measurement reflects the surface potential distribution of β-Ga_2_O_3_ films by measuring the capacitance between the probe and the surface. During test, electrons or holes were injected into β-Ga_2_O_3_ by applying -5 V bias (Fig. S16a) or 5 V bias (Fig. S16b). Within the target regions (5×5 μm), extrinsic charge carriers (electrons or holes) were introduced exclusively into the central areas (1×5 μm^2^).


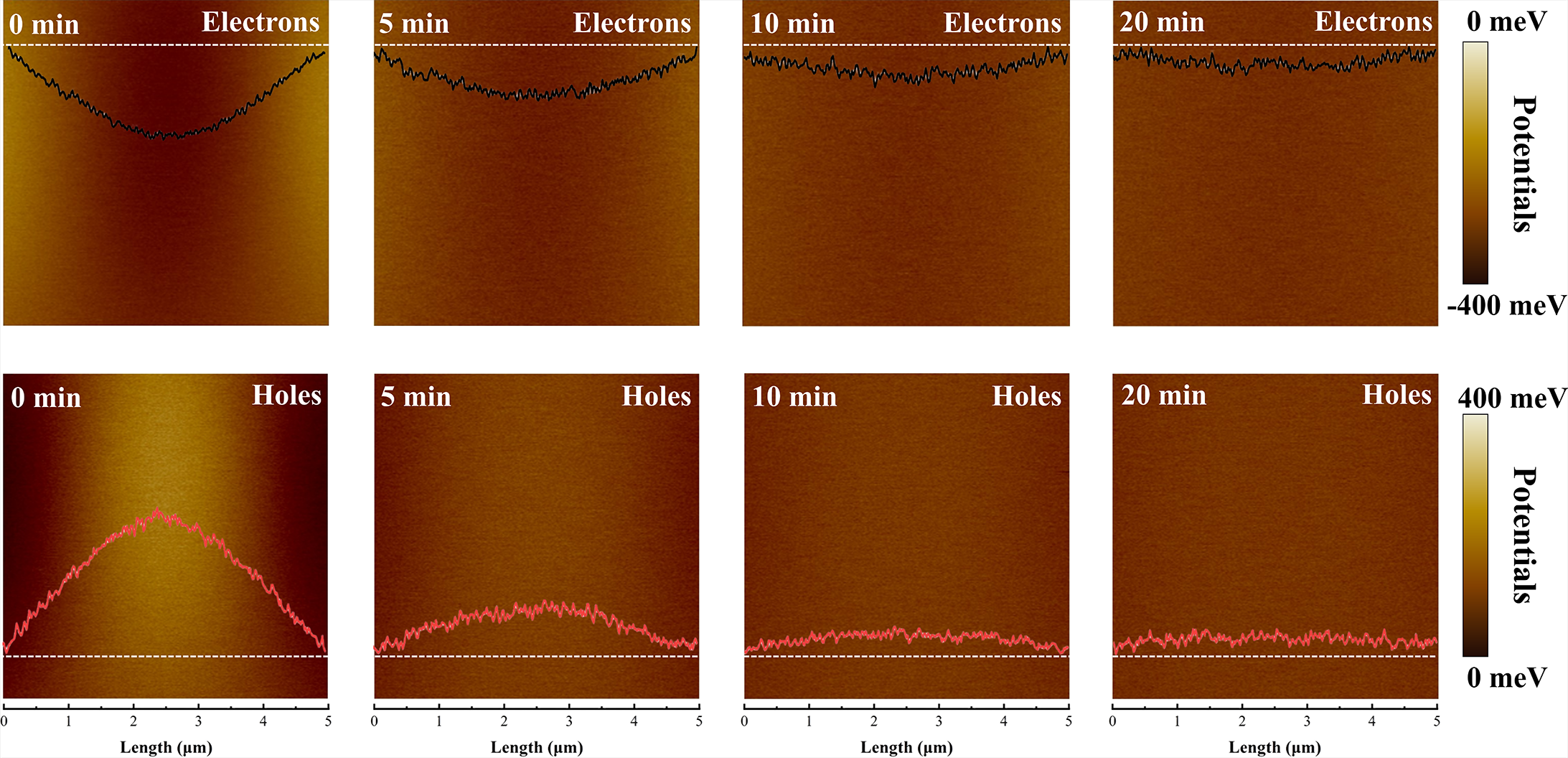


**Fig. S17 KPFM measurement on β-Ga_2_O_3_ film grown under 60 W.**

KPFM measurement of β-Ga_2_O_3_ film grown under 60 W is shown in Fig. S17. The difference in $m_{e}^{*}$ and $m_{h}^{*}$ of β-Ga_2_O_3_ led to the various surface potentials of holes at 0 min. However, after 10-minute decay, the surface potentials of holes and electrons reduced to the baseline, in contrast with that of β-Ga_2_O_3_ film grown under 120 W in Fig. 3f, indicating the PPC effect of β-Ga_2_O_3_ film grown under 120 W resulted from STHs.


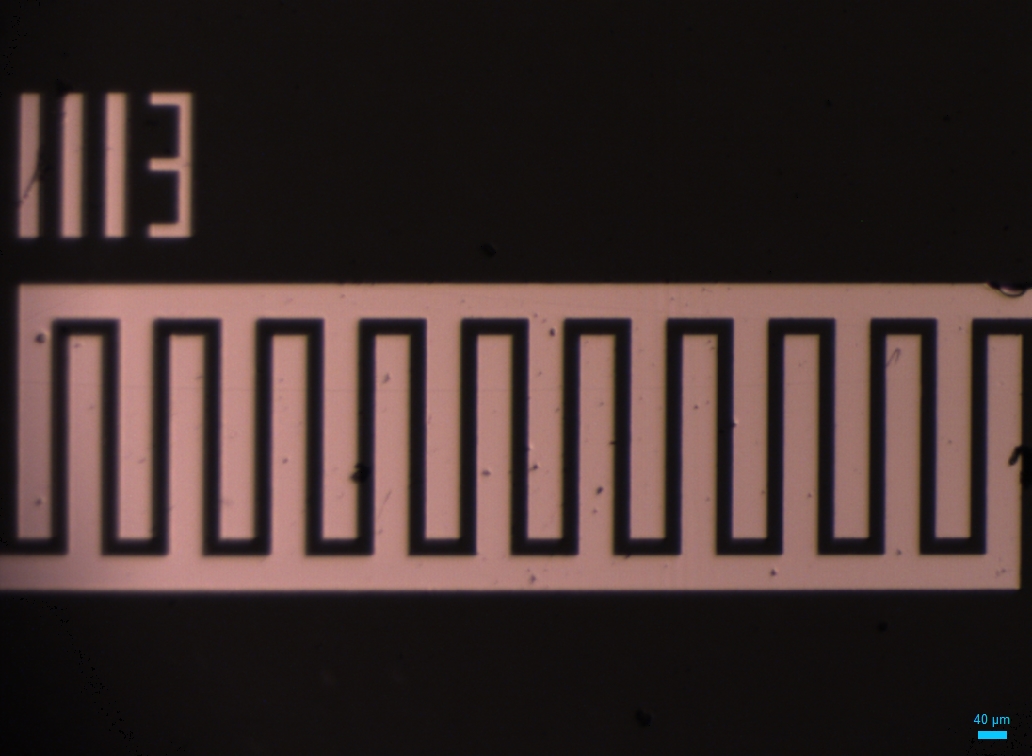


**Fig. S18 The device structure of β-Ga_2_O_3_ photo-synapses by optical microscope**


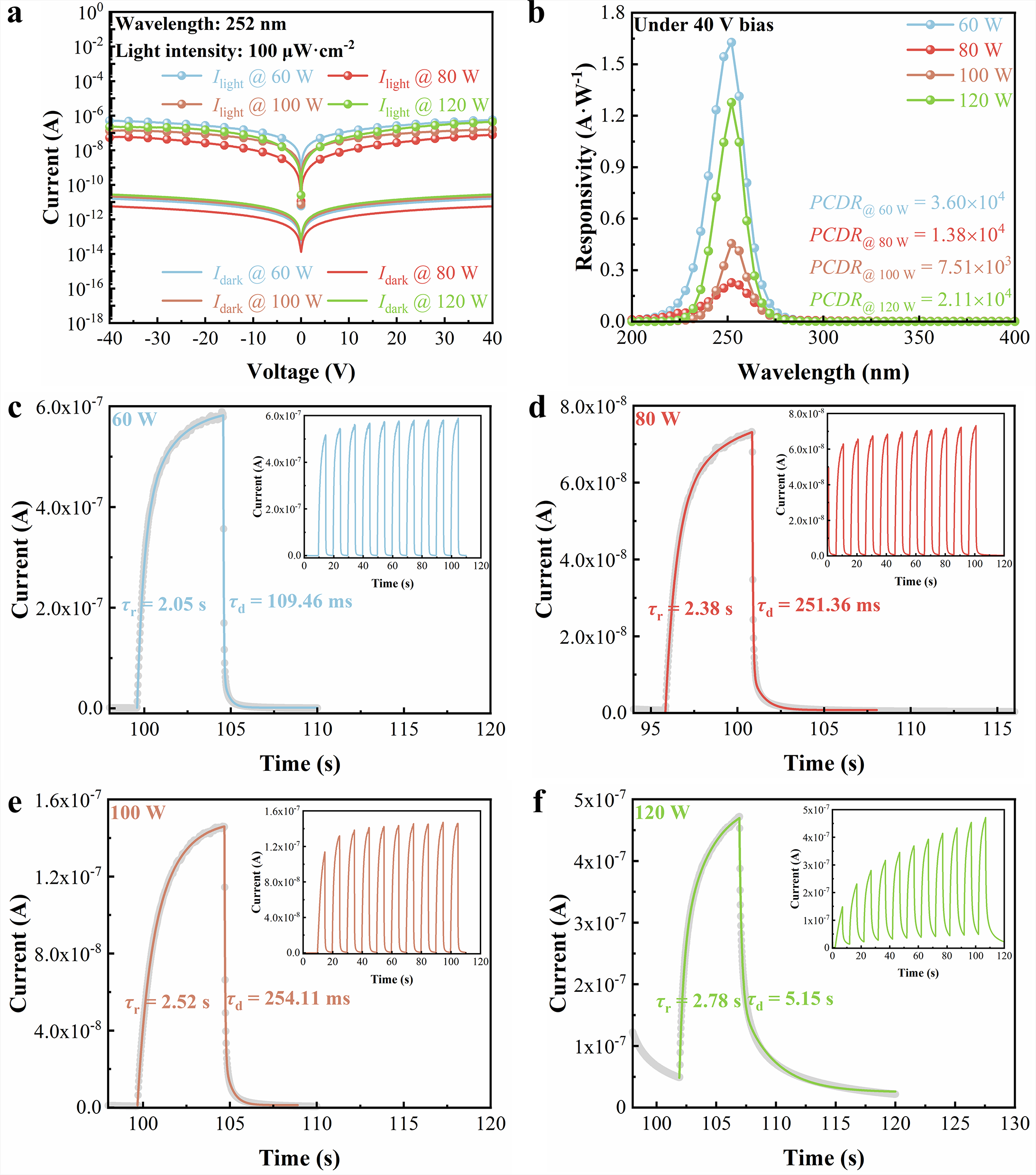


**Fig. S19 Photodetector performance of β-Ga_2_O_3_ films grown under various sputtering power. a** *I*-*V* curves. **b** responsivity. *I*-*t* curves of β-Ga_2_O_3_ films grown under **c** 60 W, **d** 80 W, **e** 100 W and **f** 120 W.

To characterize the detective performance of β-Ga_2_O_3_ films, *I*-*V* and *I*-*t* measurements were carried out, and the results are presented in Fig. S19. As shown in Fig. S19a, β-Ga_2_O_3_ films fabricated under various sputtering powers all demonstrated excellent response to 252 nm solar-blind ultraviolet light. Under 40 V bias, the response peaks of all samples consistently appear at 252 nm, indicating that variations in concentration of STHs did not alter the spectral response range. The *I*-*t* curves of β-Ga_2_O_3_ (40 V, 252 nm, 100 μW·cm^-2^) were displayed in Fig. S19 c-f. The results revealed that with increasing STHs concentration, the rising time (*τ*_r_) increased from 2.05 s to 2.78 s, representing the increase of 35.61 %. Meanwhile, the decay time (*τ*_d_) increased dramatically from 109.46 ms to 5.15 s, corresponding to the increase of 4604.92 %. Notably, a pronounced PPC effect was observed in β-Ga_2_O_3_ fabricated at 120 W, suggesting that STHs predominantly influenced the decay time, with a relatively minor effect on the rising time. These findings indicated that β-Ga_2_O_3_ photo-synapses via STHs engineering exhibited prominent EPSC characteristics.


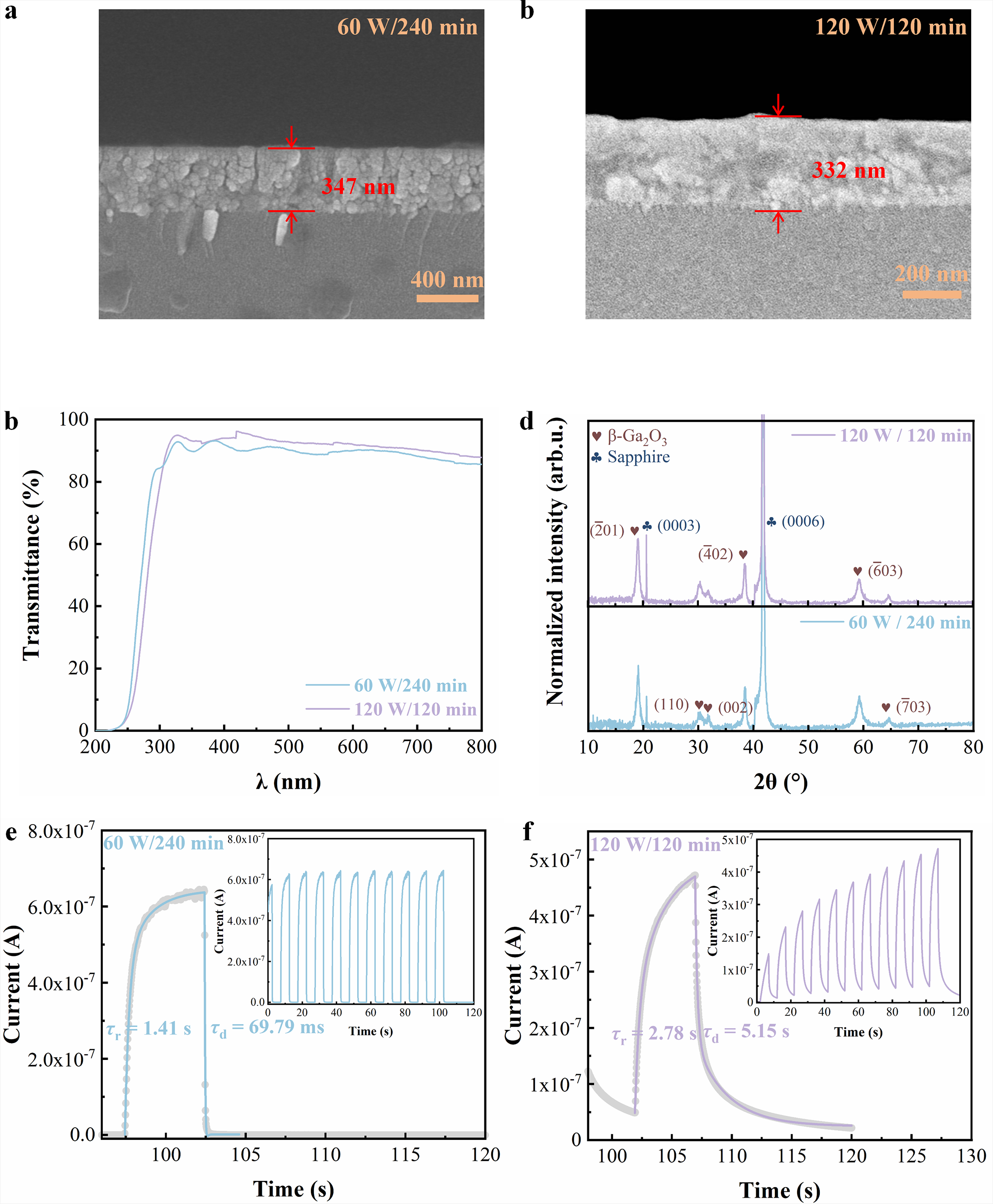


**Fig. S20 Comparison of β-Ga_2_O_3_ films with matched thickness.** Thickness of β-Ga_2_O_3_ films grown under **a** 60 W/240 min, **b** 120 W/120 min. **c** Transmittance of β-Ga_2_O_3_ films grown under 60 W/240 min and 120 W/120 min. **d** XRD of β-Ga_2_O_3_ films grown under 60 W/240 min and 120 W/120 min. *I*-*t* curves of β-Ga_2_O_3_ films grown under **e** 60 W/240 min, **f** 120 W/120 min.

Since the thickness of β-Ga_2_O_3_ films in Fig. S10 depended on sputtering power, it is essential to decouple the effect of film thickness from that of sputtering power on the concentration of STHs. To isolate the influence of thickness, the β-Ga_2_O_3_ film was deposited at 60 W/240 min to match the thickness of the reference film prepared at 120 W/120 min. As shown in Fig. S20a-b, it is clear that β-Ga_2_O_3_ films grown under 60 W/240 min achieved the thickness comparable to that of the reference films deposited at 120 W/120 min. The matched film thicknesses of two samples resulted in overlapping transmittance spectra in Fig. S20c. XRD analysis further revealed that two samples exhibited the $\text{(}\bar{\text{2}}\text{01)}$ preferred orientation and displayed three additional diffraction peaks corresponding to (110), (002) and $\text{(}\bar{\text{7}}\text{03)}$ planes, as shown in Fig. S20d. Subsequently, Ti/Au interdigital electrodes were fabricated on two samples via conventional photolithography, and the device architecture was illustrated in Fig. S18. *I-t* characteristics of the two devices were measured under identical illumination conditions (40 V, 252 nm, 100 μW·cm^-2^). As shown in Fig. S20e, β-Ga_2_O_3_ film deposited at 60 W/240 min did not exhibit PPC effect with *τ*_r_ = 1.41 s and *τ*_d_ = 69.79 ms. In contrast, β-Ga_2_O_3_ film deposited at 120 W/120 min displayed the pronounced PPC effect with *τ*_r_ = 2.78 s and *τ*_d_ = 5.15 s in Fig. S20f. The results have indicated that the variation in concentration of STHs resulted from sputtering power rather than film thickness.


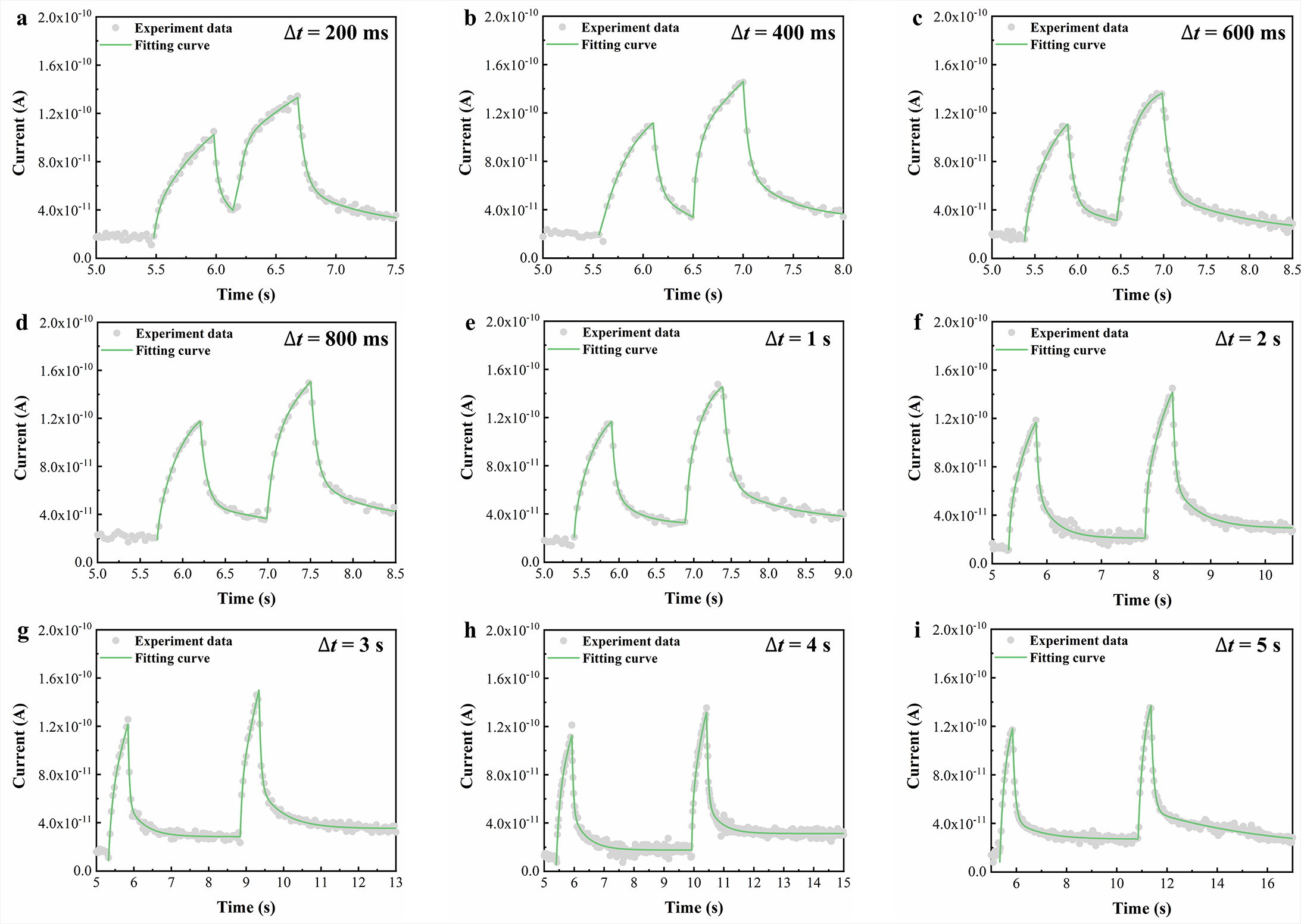


**Fig. S21 The *I-t* curves of PPF with various ∆*t*.** **a** ∆*t* = 200 ms. **b** ∆*t* = 400 ms. **c** ∆*t* = 600 ms. **d** ∆*t* = 800 ms. **e** ∆*t* = 1 s. **f** ∆*t* = 2 s. **g** ∆*t* = 3 s. **h** ∆*t* = 4 s. **i** ∆*t* = 5 s.





**Fig. S22 The *I-t* curve of learning-experiencing behavior.**





**Fig. S23 The normalized long-term conductance under UV optical potentiations and electronic depressions.**

To calculate the weight update nonlinearity, the conductance values obtained over six cycles were subjected to orthogonal normalization, as presented in Fig. S23.


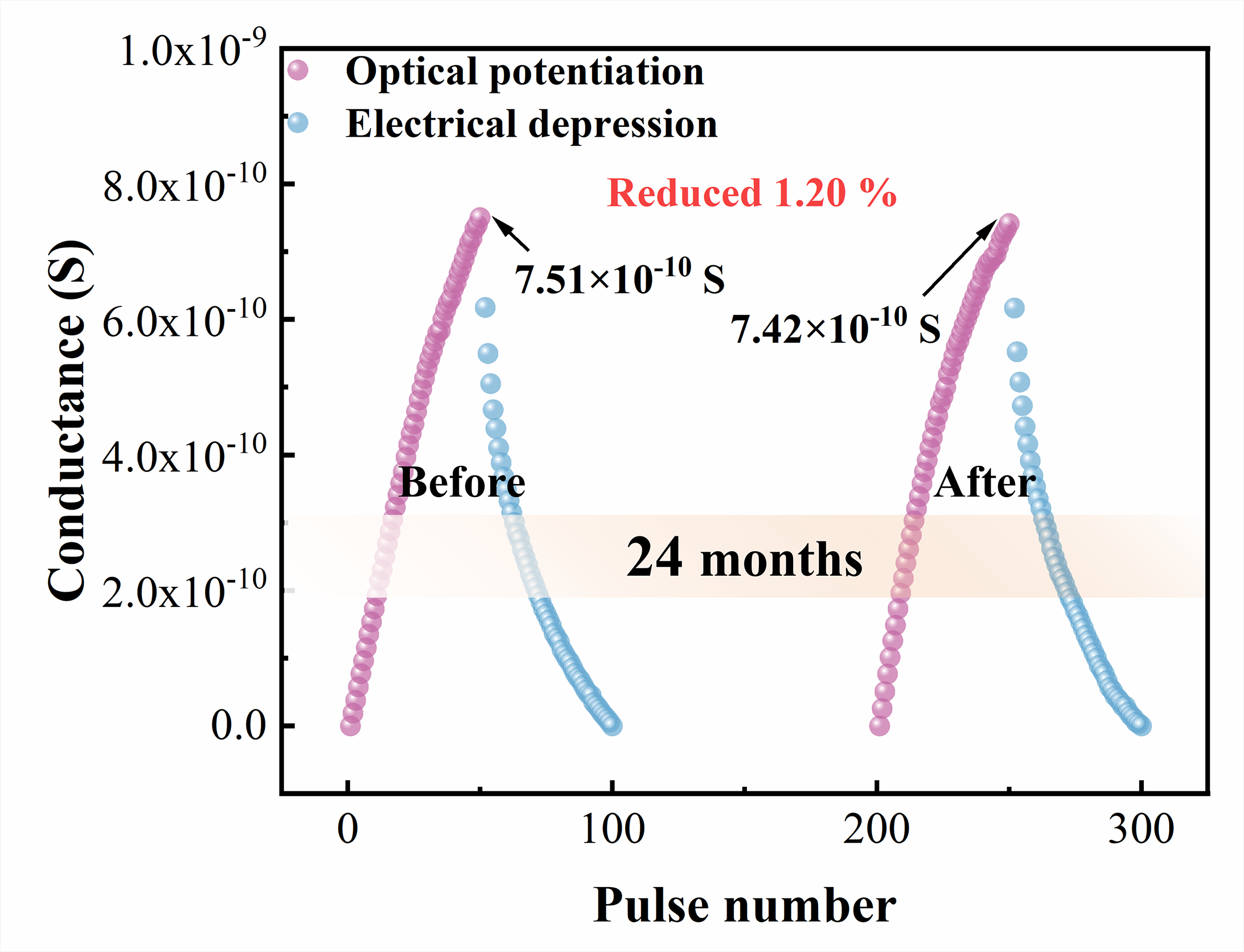


**Fig. S24 Comparison of conductance of β-Ga_2_O_3_ photo-synapses before and after 24 months of storage under atmospheric conditions.**





**Fig. S25 The *I-t* curve of learning-experiencing behavior belong to 8 β-Ga_2_O_3_ photo-synapses**

To evaluate the reproducibility of β-Ga_2_O_3_ photo-synapses in this work, eight β-Ga_2_O_3_ photo-synapses were fabricated by using the same fabrication processes, and their learning-experience characteristics were systematically characterized. As illustrated in Fig. S25, all devices exhibited robust learning-experience behavior, demonstrating the reproducibility of β-Ga_2_O_3_ photo-synapses by STHs engineering.


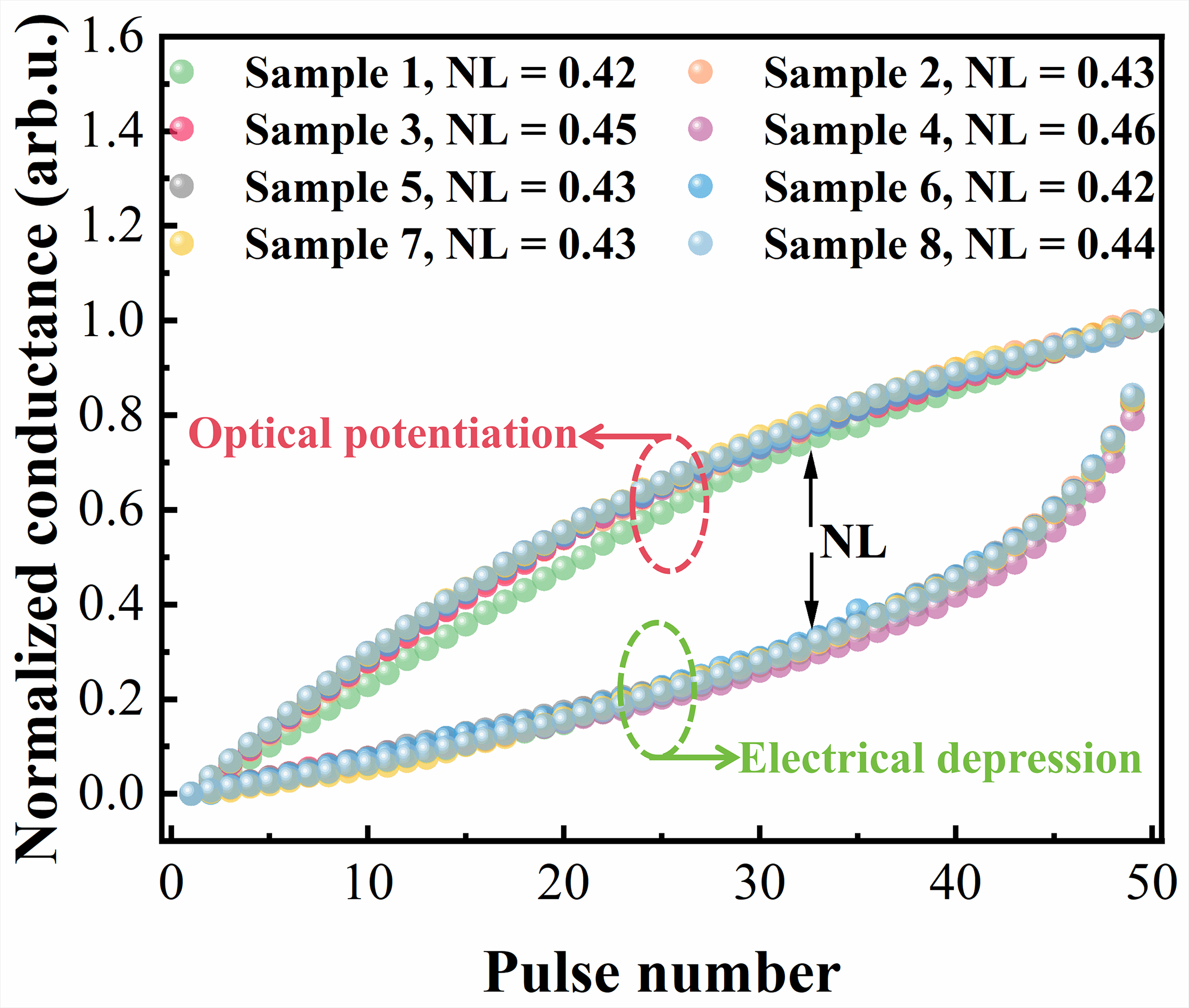


**Fig. S26 Weight update nonlinearity (NL) belong to 8 β-Ga_2_O_3_ photo-synapses.**

To evaluate the reproducibility of β-Ga_2_O_3_ photo-synapses in this work, eight β-Ga_2_O_3_ photo-synapses were fabricated using the same fabrication processes, and their weight update nonlinearity (NL) were systematically characterized. As shown in Fig. S26, the value of NL ranged from 0.42 to 0.46, demonstrating the reproducibility of β-Ga_2_O_3_ photo-synapses by STHs engineering.


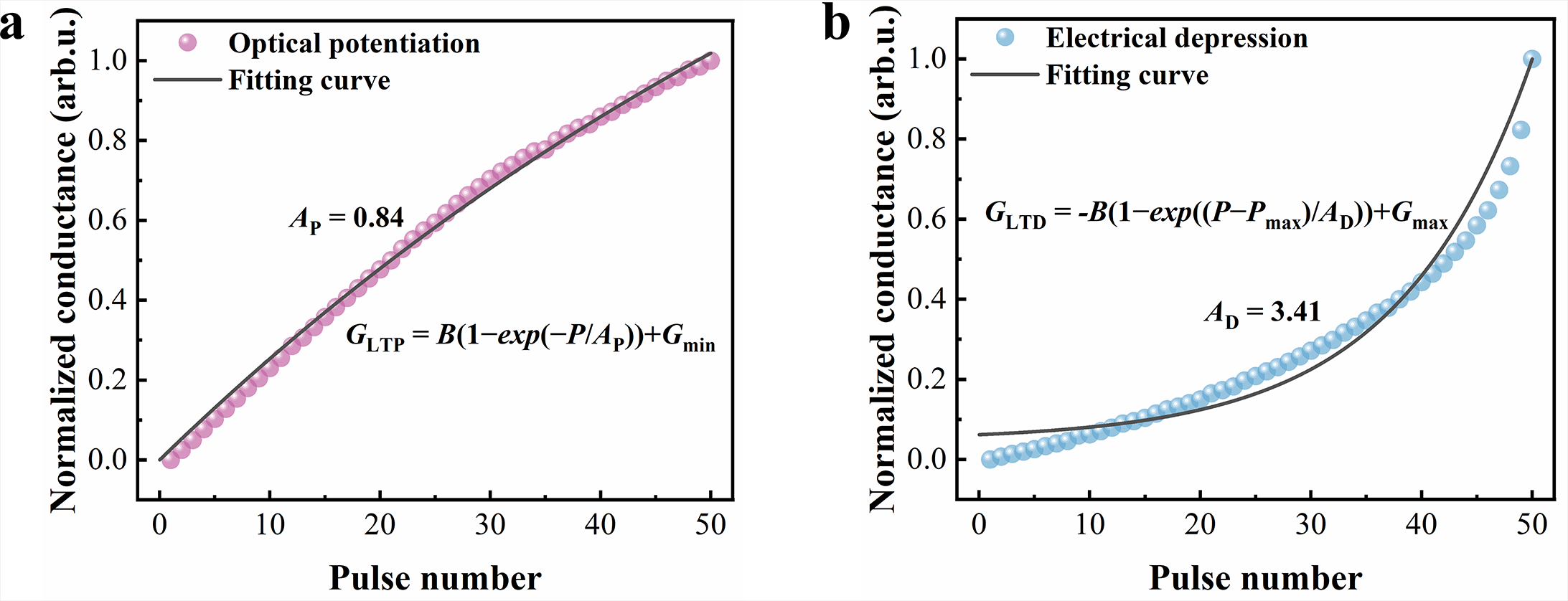


**Fig. S27 Nonlinear normalized conductivity of β-Ga_2_O_3_ photo-synapses. a** LTP. **b** LTD.

For β-Ga_2_O_3_ photo-synapses, the weight update nonlinearity can also be determined based on the alternative approach proposed by Matthew Jerry et al., as follow^24^:

$\text{G}_{\text{LTP}}\text{=}\text{B}\text{(1−}\text{exp}\text{(−}\frac{\text{P}}{\text{A}_{\text{P}}}\text{))+}\text{G}_{\text{min}}$ **(Equation S10)**

$\text{G}_{\text{LTD}}\text{=−}\text{B}\text{(1−}\text{exp}\text{(}\frac{\text{P}\text{−}\text{P}_{\text{max}}}{\text{A}_{\text{D}}}\text{))+}\text{G}_{\text{max}}$ **(Equation S11)**

$\text{B}\text{=}\frac{\text{G}_{\text{max}}\text{−}\text{G}_{\text{min}}}{\text{1−}\text{exp}\text{(−}\frac{\text{P}_{\text{max}}}{\text{A}_{\text{P,D}}}\text{)}}$ **(Equation S12)**

Where $\text{G}_{\text{max}}$ and $\text{G}_{\text{min}}$are the maximum and minimum conductivity values of β-Ga_2_O_3_ photo-synapses. $\text{P}_{\text{max}}$ signifies the maximum pulse number. $\text{A}_{\text{P}}$ and $\text{A}_{\text{D}}$ are the weight update nonlinearity, $\text{B}$ is simply a function of $\text{A}_{\text{P}}$ and $\text{A}_{\text{D}}$. In this work, $A_{\text{P}}$ = 0.84 and $A_{\text{D}}$ = 3.41.


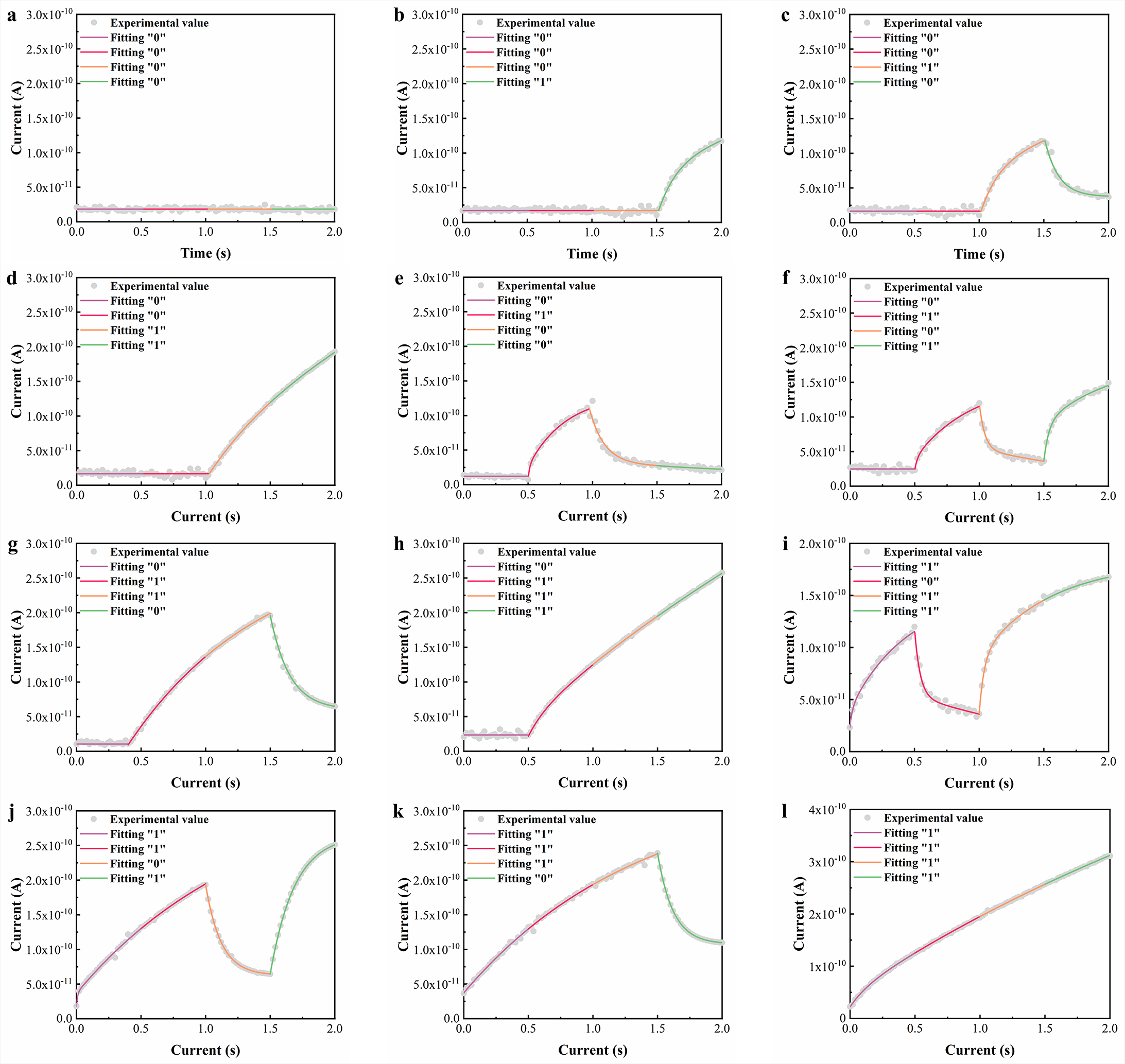


**Fig. S28 4-bit *I-t* curves of β-Ga_2_O_3_ photo-synapses. a** 0000. **b** 0001. **c** 0010. **d** 0011. **e** 0100. **f** 0101. **g** 0110. **h** 0111. **i** 1011. **j** 1101. **k** 1110. **l** 1111.

**Table S1** Thermodynamic transition levels of intrinsic defects in β-Ga_2_O_3_

| Defects | Thermodynamic transition levels | Our work (eV; above VBM) | Defects | Thermodynamic transition levels | Our work (eV; above VBM) |
| --- | --- | --- | --- | --- | --- |
| $\text{V}_{\text{Ga}_{\text{Ⅰ}}}$ | (2-/3-) | 3.13 | $\text{STHs}_{\text{@}\text{O}_{\text{Ⅰ}}}$ | (+/0) | 0.56 |
|  | (-/2-) | 2.56 |  |  |  |
|  | (0/-) | 2.06 |  |  |  |
|  | (+/0) | 1.57 |  |  |  |
| $\text{V}_{\text{Ga}_{\text{Ⅱ}}}$ | (2-/3-) | 2.86 |  |  |  |
|  | (-/2-) | 2.35 |  |  |  |
|  | (0/-) | 1.87 |  |  |  |
|  | (+/0) | 1.47 |  |  |  |
| $\text{V}_{\text{O}_{\text{Ⅰ}}}$ | (2+/0) | 3.29 | $\text{STHs}_{\text{@}\text{O}_{\text{Ⅲ}}}$ | (+/0) | 0.54 |
|  | (2+/+) | 3.48 |  |  |  |
|  | (+/0) | 3.10 |  |  |  |
| $\text{V}_{\text{O}_{\text{Ⅱ}}}$ | (2+/0) | 2.70 |  |  |  |
|  | (2+/+) | 2.94 |  |  |  |
|  | (+/0) | 2.46 |  |  |  |
| $\text{V}_{\text{O}_{\text{Ⅲ}}}$ | (2+/0) | 3.44 | O*_i_* | (+/0) | 1.58 |
|  | (2+/+) | 3.72 |  |  |  |
|  | (+/0) | 3.17 |  |  |  |

The formation energies and thermodynamic transition levels of $\text{V}_{\text{Ga}_{\text{Ⅰ}}}$、$\text{V}_{\text{Ga}_{\text{Ⅱ}}}$、$\text{V}_{\text{O}_{\text{Ⅰ}}}$、$\text{V}_{\text{O}_{\text{Ⅱ}}}$、$\text{V}_{\text{O}_{\text{Ⅲ}}}$、O*_i_*、$\text{STHs}_{\text{@}\text{O}_{\text{Ⅲ}}}$ were calculated in our previous researches^14,15^ and re-presented in Fig. 2 and Table S1.

**Table S2** Sputtering deposition parameters for β-Ga_2_O_3_ films

| Sputtering power (W) | O_2_ flow rate (SCCM) | Ar flow rate (SCCM) | Sputtering pressure (Pa) | Sputtering time (min) | Annealing temperature (℃) | Annealing time (min) |
| --- | --- | --- | --- | --- | --- | --- |
| 60 | 4 | 40 | 1.4 | 120 | 800 | 120 |
| 80 |  |  |  |  |  |  |
| 100 |  |  |  |  |  |  |
| 120 |  |  |  |  |  |  |

**Table S3** Ga and O atoms of β-Ga_2_O_3_ film grown under various sputtering power

| Sputtering power (W) | Ga atom (%) | O atom (%) |
| --- | --- | --- |
|  |  |  |
| 60 | 37.41 | 62.59 |
| 80 | 38.29 | 61.71 |
| 100 | 38.39 | 61.61 |
| 120 | 38.81 | 61.19 |

**Table S4** O_Ⅰ_ and O_Ⅱ_ of β-Ga_2_O_3_ O 1*s* peaks under various sputtering powers

| Sputtering power (W) | O_Ⅰ_ (%) | O_Ⅱ_ (%) |
| --- | --- | --- |
| 60 | 66.68 | 33.32 |
| 80 | 67.48 | 32.52 |
| 100 | 66.04 | 33.96 |
| 120 | 66.52 | 33.48 |

**Table S5** The peak percentage of β-Ga_2_O_3_ films

| Sputtering Power (W) | Peak area percentage (%) | | |
| --- | --- | --- | --- |
|  | UVL | BL+GL | RL |
| 60 | 2.74 | 64.48 | 32.78 |
| 80 | 4.31 | 64.47 | 31.22 |
| 100 | 4.14 | 65.29 | 30.57 |
| 120 | 4.45 | 65.27 | 30.28 |

**Table S6** Interdigital electrode parameters of β-Ga_2_O_3_ photo-synapses

| Parameter (μm) | | | |
| --- | --- | --- | --- |
| Length (*l*) | Width (*w*) | Spacing (*d*) | Active area (cm^2^) |
| 300 | 50 | 200 | 0.0034 |

**Table S7** Energy consumption of per synaptic event for β-Ga_2_O_3_ photo-synapses

| Function | *P*_spike_ (μW·cm^-2^) | *t*_duration_ (s) | *E*_potentiation_ (J·µm^-2^) | *V*_read_ (V) | *J*_out_ (A·µm^-2^) (average) | *T*_duration_ (s) | *E*_depression_ (J·µm^-2^) (average) |
| --- | --- | --- | --- | --- | --- | --- | --- |
| SRDP | 66.2 | 0.5 | 3.31×10^-13^ | \ | \ | \ | \ |
| SIDP | 123.8 | 0.5 | 6.19×10^-13^ | \ | \ | \ | \ |
| STDP | 66.2 | 20 | 1.32×10^-11^ | \ | \ | \ | \ |
| SFDP | 66.2 | 0.5 | 3.31×10^-13^ | \ | \ | \ | \ |
| Learning-experience behavior | 66.2 | 0.5 | 3.31×10^-13^ | \ | \ | \ | \ |
| Long-term conductance | 66.2 | 0.5 | 3.31×10^-13^ | -10 | 1.25×10^-6^ | 0.5 | 6.25×10^-6^ |

The estimation of energy consumption for β-Ga_2_O_3_ photo-synapses is based on the following equations^25-29^:

$\text{E}_{\text{potentiation}}\text{=}\text{P}_{\text{spike}}\text{×}\text{t}_{\text{duration}}$ **(Equation S13)**

$\text{E}_{\text{depression}}\text{=}\text{V}_{\text{read}}\text{×}\text{J}_{\text{out}}\text{×}\text{T}_{\text{duration}}$ **(Equation S14)**

Where $\text{E}_{\text{potentiation}}$ and $\text{E}_{\text{depression}}$ denote the energy consumption in a single optical potentiation process and electrical depression process. $\text{P}_{\text{spike}}$, and $\text{t}_{\text{duration}}$ are the optical spike power and the spike duration, respectively. $\text{V}_{\text{read}}$, $\text{J}_{\text{out}}$ and $\text{T}_{\text{duration}}$ represent read voltage, output current density and electronic depression duration. SRDP function is the spike-rate-dependent plasticity in Fig. 4d. SIDP function is the spike-intensity-dependent plasticity function in Fig. 4e. STDP function is spike-time-dependent plasticity in Fig. 4f. SFDP function is spike-frequency dependent plasticity in Fig. 4g. Learning-experience behavior is shown in Fig. 4h and long-term conductance function under UV optical potentiations and electronic depressions is shown in Fig. 4i.

**Table S8** Detecting performance of various photo-synapses

| Photo-synapses | *R*  (A·W^-1^) | *D*^*^ (Jones) | Weight update nonlinearity | Energy consumption of per optical potentiation (J·µm^-2^) | Reference |
| --- | --- | --- | --- | --- | --- |
| Ga_2_O_3_/MoS_2_ | 0.029 | 1.3×10^9^ | \ | \ | Ref. 25 |
| GaN/Ga_2_O_3_/GaN | 0.004 | 1.13×10^13^ | \ | 5.59×10^-13^ | Ref. 26 |
| MoS_2_/WS_2_/h-BN | 9.28 | 6.1×10^12^ | \ | ~1×10^-12^ | Ref. 27 |
| WSe_2_/ZnO | 7000 | 8×10^12^ | \ | 2.27×10^-10^ | Ref. 28 |
| Ag/Ga_2_O_3_/Pt | \ | \ | 0.72 | 1.20×10^-11^ | Ref. 29 |
| Ag NPs/WO_x_ | \ | \ | 0.4 | 4×10^-9^ | Ref. 30 |
| Bi_2_O_2_Se/graphene | 110 | \ | \ | 3.1×10^-12^ | Ref. 31 |
| Zn_2_SnO_4_/MgO | \ | \ | ~0.44 | 2×10^-8^ | Ref. 32 |
| MXene/Y:HfO_2_ | \ | \ | ~0.78 | 8.3×10^-11^ | Ref. 33 |
| MoS_2_/SiO_2_/p-Ge | 0.55 | \ | \ | 6.35×10^-11^ | Ref. 34 |
| WO_3–x_/WO_3–x_-Ag/WO_3–x_ | \ | \ | 0.73 | 5×10^-12^ | Ref. 35 |
| SnSe/InSe/  GaN | 1.1 | 1.86×10^13^ | \ | 2.54×10^-10^ | Ref. 36 |
| Sb_2_S_3_ | \ | \ | 0.65 | \ | Ref. 37 |
| β-Ga_2_O_3_ | 1.28 | 2.86×10^13^ | 0.42 | 3.31×10^-13^ | This work |

For β-Ga_2_O_3_ photo-synapses, the performance remain stable under ultraviolet irradiation, indicating no observable degradation over time. The evaluation parameters of detecting performance are similar to those of photodetectors, including responsivity (*R*) and detectivity (*D*^*^), which can be calculated using the following equations^38^:

$\text{R}\text{=}\frac{\text{EQE}\text{·}\text{e}}{\text{h}\text{v}}\text{=}\frac{\text{I}_{\text{ph}}}{\text{P}_{\text{in}}}\text{=}\frac{\text{I}_{\text{light}}\text{−}\text{I}_{\text{dark}}}{\text{ρ}_{\text{in}}\text{·}\text{S}}$ **(Equation S15)**

*D*^*^$\text{=}\frac{\text{R}}{\sqrt{\text{2}\text{e}\text{J}_{\text{d}}}}$ **(Equation S16)**

Where $\text{ρ}_{\text{in}}$ is incident light power density, *S* is the active area and $\text{I}_{\text{ph}}$ is the difference that the photocurrent (*I*_light_) minus the dark current (*I*_dark_), $\text{R}$ is the responsivity, $\text{e}$ is the electron charge, $\text{J}_{\text{d}}$ is the dark current density. $\text{R}$ quantifies the sensitivity of β-Ga_2_O_3_ photo-synapses to incident illumination. *D*^*^ reflects the ability to distinguish a signal from background noise and serves as a key metric for evaluating the signal-to-noise ratio.

The computing performance can be evaluated based on weight update nonlinearity and energy consumption per optical potentiation. The weight update nonlinearity reflects the linearity of synaptic weight modulation and has been quantitatively analyzed in the manuscript, where a lower value indicates superior linear computational behavior. The energy consumption per optical potentiation, which characterizes the operational energy efficiency of the device, is comprehensively detailed in Table S7.

**Table S9** Synaptic performance of β-Ga_2_O_3_ photo-synapses

| Photo-synapses | Working mechanism | Rising time (s) | Decay time (s) | Weight update nonlinearity | Stability | Reference |
| --- | --- | --- | --- | --- | --- | --- |
| Ag/Ga_2_O_3_/Pt | *V*_O_ | ~ 1.75 s _@ 3 s illumination_ | > 15 s _@ 3 s illumination_ | 0.72 | \ | Ref. 29 |
| Zn:Ga_2_O_3_ | *V*_O_ | ~ 2.82 s _@ 3 s illumination_ | ~50 s _@ 3 s illumination_ | 0.59 | \ | Ref. 39 |
| Zn_2_SnO_4_/Ga_2_O_3_ | *V*_O_ | ~ 78.08 s _@ 100 s illumination_ | 775 s _@ 100 s illumination_ | 0.48 | \ | Ref. 40 |
| Pt/Ga_2_O_3_/Pt | *V*_O_ | ~ 1.8 s _@ 3 s illumination_ | > 48 s _@ 3 s illumination_ | 0.76 | \ | Ref. 41 |
| β-Ga_2_O_3_/ZnO | *V*_O_ | ~ 0.62 s _@ 0.5 s illumination_ | > 17 s _@ 0.5 s illumination_ | 0.80 | \ | Ref. 42 |
| Ga_2_O_3_/ZnO | *V*_O_ | ~ 1.86 s _@ 2 s illumination_ | > 9 s _@ 2 s illumination_ | \ | \ | Ref. 43 |
| β-Ga_2_O_3_ | *V*_O_ | ~ 2.54 s _@ 3 s illumination_ | > 28 s _@ 3 s illumination_ | \ | \ | Ref. 44 |
| β-Ga_2_O_3_ | STHs | 0.40 s _@ 0.5 s illumination_  1.68 s _@ 2 s illumination_ | > 10 s _@ 0.5 s illumination_  > 10 s _@ 2 s illumination_ | 0.42 | 24 Months | This work |

The rising time, decay time, weight update nonlinearity and stability of β-Ga_2_O_3_ photo-synapse in this work were systematically compared with those based on *V*_O_ strategy, as summarized in Table S9. Our β-Ga_2_O_3_ photo-synapse exhibited faster rising time. In contrast, β-Ga_2_O_3_ photo-synapses based on *V*_O_ strategy displayed prolonged decay time and PPC effect, indicating superior data retention and hallmarks of nonvolatile memory behavior. As illustrated in Fig. S1, the STH-engineered β-Ga_2_O_3_ photo-synapses operated via the charge trapping mechanism where synaptic behavior resulted from hole-trapping at STHs, without involvement of ion migration and formation of conductive filament. Consequently, our β-Ga_2_O_3_ photo-synapses achieved rapid response speed and lower weight update nonlinearity, suitable for volatile in-sensor computing. By contrast, β-Ga_2_O_3_ photo-synapses based on *V*_O_ strategy functioned as conductive filamentary devices, where synaptic behavior stemmed from ionization and migration of *V*_O_. Due to the inherently slower ionic motion compared to carrier transport, such devices suffer from sluggish response speeds and pronounced weight update nonlinearity. Nevertheless, their distinct PPC effect enabled long-term charge retention, conferring intrinsic non-volatility and making them better suited for in-memory computing.

**Table S10** Tracking accuracy of β-Ga_2_O_3_ photo-synapses under various Gaussian noise levels

| Gaussian noise levels (%) | Tracking accuracy (%) |
| --- | --- |
| 10 | 100 |
| 20 | 100 |
| 30 | 100 |
| 40 | 100 |
| 50 | 100 |
| 60 | 100 |
| 70 | 88.3 |
| 80 | 54.5 |
| 90 | 28.6 |

References

1. Pan, Y. X. et al. Effects of hydration and oxygen vacancy on CO_2_ adsorption and activation on β-Ga_2_O_3_ (100). *Langmuir* **26**, 5551–5558 (2010).
2. Pan, Y. X. et al. Photocatalytic CO_2_ reduction highly enhanced by oxygen vacancies on Pt-nanoparticle-dispersed gallium oxide. *Nano Research.* **9**, 1689-1700 (2016).
3. Teramura, K. et al. Effect of H_2_ gas as a reductant on photoreduction of CO_2_ over a Ga_2_O_3_ photocatalyst. *Chemical Physics Letters* **467**, 191-194 (2008).
4. Liu, T. F. et al. Role of oxygen vacancies on oxygen evolution reaction activity: β-Ga_2_O_3_ as a case study. *Chemistry of Materials.* **30**, 7714-7726 (2018).
5. Chang, M. M. et al. Impact of 100 MeV high-energy proton irradiation on β-Ga_2_O_3_ solar-blind photodetector: oxygen vacancies formation and resistance switching effect. *Journal of Applied Physics* **132**, 123105 (2022).
6. Guo, D. Y. et al. Oxygen vacancy tuned Ohmic-Schottky conversion for enhanced performance in β-Ga_2_O_3_ solar-blind ultraviolet photodetectors. *Applied Physics Letters* **105**, 023507 (2014).
7. Varley, J. B. et al. Role of self-trapping in luminescence and p-type conductivity of wide-band-gap oxides. *Physical Review B* **85**, 081109(R) (2012).
8. Frodason, Y. K. Self-trapped hole and impurity-related broad luminescence in β-Ga_2_O_3_. *Journal of Applied Physics* **127**, 075701 (2020).
9. Whalley, L. D. et al. Impact of nonparabolic electronic band structure on the optical and transport properties of photovoltaic materials. *Physical Review B* **99**, 085207 (2019).
10. Mohamed, M. et al. The electronic structure of β-Ga_2_O_3_. *Applied Physics Letters* **97**, 211903 (2010).
11. Janowitz, C. et al. Experimental electronic structure of In_2_O_3_ and Ga_2_O_3_, *New Journal of Physics* **13**, 085014 (2011).
12. Reshchikov, M. A. et al. Evaluation of the concentration of point defects in GaN. *Scientific Reports* **7**, 9297 (2017).
13. Wang, X. T. et al. Bio-inspired optoelectronic devices and systems for energy-efficient in-sensor computing. *npj Unconventional Computing* **2**, 15 (2025).
14. Nie, Y. Y. et al. Modulating the blue and green luminescence in the β-Ga_2_O_3_ films. *Journal of Alloys and Compounds* **900**, 163431 (2022).
15. Nie, Y. Y. et al. Achieving ultra-low dark current in β-Ga_2_O_3_ photoconductive photodetectors for anti-interference optical human-machine interaction systems via gallium interstitials engineering. *Small* **21**, 2501442 (2025).
16. Varley, J. B. et al. Role of self-trapping in luminescence and p-type conductivity of wide-band-gap oxides. *Physical Review B* **85**, 081109(R) (2012).
17. Gake, T. et al. First-principles study of self-trapped holes and acceptor impurities in Ga_2_O_3_ polymorphs. *Physical Review Materials* **3**, 044603 (2019).
18. Frodason, Y. K. et al. Self-trapped hole and impurity-related broad luminescence in β-Ga_2_O_3_. *Journal of Applied Physics* **127**, 075701 (2020).
19. Traiwattanapong, W. et al. Self-trapped holes in BaTiO_3_, *Journal of Applied Physics* **124**, 085703 (2018).
20. Deskins, N. A. et al. Electron transport via polaron hopping in bulk TiO_2_: A density functional theory characterization. *Physical Review B* **75**, 195212 (2007).
21. Sicolo, S. et al. Structure and ESR properties of self-trapped holes in pure silica from first-principles density functional calculations. *Physical Review B* **76**, 075121 (2007).
22. Nie, Y. Y. et al. Growth and properties analysis of Al_x_Ga_2-x_O_3_ thin film by radio frequency magnetron sputtering using Al/Ga_2_O_3_ target. *Journal of Alloys and Compounds* **798**, 568-575 (2019).
23. Li, S. F. et al. The influence of sputtering power on the structural, morphological and optical properties of β-Ga_2_O_3_ thin films. *Journal of Alloys and Compounds* **753**, 186-191 (2018).
24. Jerry, M. et al. Ferroelectric FET analog synapse for acceleration of deep neural network training. *2017 IEEE International Electron Devices Meeting (IEDM)*, 6.2.1-6.2.4 (San Francisco, CA, USA, 2017).
25. Zhang, Y. et al. Optoelectronic neuromorphic logic memory device based on Ga_2_O_3_/MoS_2_ Van der Waals heterostructure with high rectification and on/off ratios. *Advanced Functional Materials* **34**, 2408978 (2024).
26. Feng, S. Y. et al. Dual-mode conversion of photodetector and neuromorphic vision sensor via bias voltage regulation on a single device. *Advanced Materials* **35**, 2308090 (2023).
27. Fan, W. H. et al. Multifunction realization in MoS_2_/WS_2_/h-BN heterojunction: integrated self-powered high-performance photodetection, visualization, nonvolatile memory, and synaptic simulation. *Nano Energy* **128**, 109900 (2024).
28. Li, X. M. et al. Photovoltage junction memtransistor for optoelectronic in-memory computing. *Journal of Materials Chemistry C* **12**, 12763 (2024).
29. Cui, D. S. et al. Versatile optoelectronic memristor based on widebandgap Ga_2_O_3_ for artificial synapses and neuromorphic computing. *Light: Science & Applications* **14**, 161 (2025).
30. Cheng, Y. K. et al. Color recognition achieved in multiwavelength controlled plasmonic optoelectronic memristor for neuromorphic visual system. *Advanced Functional Materials* **35**, 2414404 (2025).
31. Yang, C. M. et al. Bidirectional all-optical synapses based on a 2D Bi_2_O_2_Se/graphene hybrid structure for multifunctional optoelectronics. *Advanced Functional Materials* **30**, 2001598 (2020).
32. Hsu, C. C. et al. ZTO/MgO-based optoelectronic synaptic memristor for neuromorphic computing. *IEEE Transactions on Electron Devices* **70**, 1048-1054 (2023).
33. Fang, J. L. et al. New-style logic operation and neuromorphic computing enabled by optoelectronic artificial synapses in an MXene/Y:HfO_2_ ferroelectric memristor. *ACS Applied Materials & Interfaces* **16**, 31348-31362 (2024).
34. Kim, S. G. et al. Infrared detectable MoS_2_ phototransistor and its application to artificial multilevel optic-neural synapse. *ACS Nano* **13**, 10294-10300 (2019).
35. Yang, W. H. et al. A network intrusion detection system with broadband WO_3-x_/WO_3-x_-Ag/WO_3-x_ optoelectronic memristor. *Advanced Functional Materials* 34, 2312885 (2024).
36. Zhang, Y. N. et al. Ultra-sensitive broadband photoresponse realized in epitaxial SnSe/InSe/GaN heterojunction for light adaptive artificial optoelectronic synapses. *Nano Energy* **133**, 110511 (2025).
37. Kundale, S. S. et al. Multilevel conductance states of vapor-transport-deposited Sb_2_S_3_ memristors achieved via electrical and optical modulation. *Advanced Science* **11**, 2405251 (2024).
38. Shi, L. L. et al. Status and outlook of metal-inorganic semiconductor-metal photodetectors. *Laser & Photonics Reviews* **15**, 2000401 (2021).
39. Fan, H. C. et al. Zn-doped Ga_2_O_3_ based two-terminal artificial synapses for neuromorphic computing applications. *Science China Materials* **68**, 3767-3777 (2025).
40. Shrivastava, S. et al. A violet-light-responsive ReRAM based on Zn_2_SnO_4_/Ga_2_O_3_ heterojunction as an artificial synapse for visual sensory and in-memory computing. *Advanced Electronic Materials* **11**, 2400527 (2025).
41. Cui, D. S. et al. Coexistence of unipolar and bipolar resistive switching in optical synaptic memristors and neuromorphic computing. *Chip* **4**, 100122 (2025).
42. Sun, S. Y. et al. Fully UV modulated artificial synapses with integrated sensing, storage and computation. *Advanced Functional Materials* **34**, 2401403 (2024).
43. Wang, J. T. et al. Piezo-phototronic effect modulated optoelectronic artificial synapse based on a-Ga_2_O_3_/ZnO heterojunction. *Nano Energy* **120**, 109128 (2024).
44. Luo, J. S. et al. Catalyst-free polymorphic β-Ga_2_O_3_ nanomaterials for solar-blind optoelectronic devices: applications in imaging and neural communication. *Small Methods* **9**, 2401473 (2025).
45. Lukoševicius, M. et al. Reservoir computing approaches to recurrent neural network training. *Computer Science Review* **3**, 127-149 (2009).
46. Tanaka, G. et al. Recent advances in physical reservoir computing: a review. *Neural Networks* **115**, 100-123 (2019).
47. Huang, H. Y. et al. Fully integrated multi-mode optoelectronic memristor array for diversified in-sensor computing. *Nature Nanotechnology* **20**, 93-103 (2025).
